# Influence of Gene Expression on Hardness in Wheat

**DOI:** 10.1371/journal.pone.0164746

**Published:** 2016-10-14

**Authors:** Ravi C. Nirmal, Agnelo Furtado, Colin Wrigley, Robert J. Henry

**Affiliations:** Queensland Alliance for Agriculture and Food Innovation, University of Queensland, Brisbane, St Lucia, Qld, Australia; Huazhong University of Science and Technology, CHINA

## Abstract

Puroindoline (*Pina and Pinb)* genes control grain texture or hardness in wheat. Wild-type/soft alleles lead to softer grain while a mutation in one or both of these genes results in a hard grain. Variation in hardness in genotypes with identical *Pin* alleles (wild-type or mutant) is known but the molecular basis of this is not known. We now report the identification of wheat genotypes with hard grain texture and wild-type/soft *Pin* alleles indicating that hardness in wheat may be controlled by factors other than mutations in the coding region of the *Pin* genes. RNA-Seq analysis was used to determine the variation in the transcriptome of developing grains of thirty three diverse wheat genotypes including hard (mutant *Pin*) and soft (wild type) and those that were hard without having *Pin* mutations. This defined the role of pin gene expression and identified other candidate genes associated with hardness. *Pina* was not expressed in hard wheat with a mutation in the *Pina* gene. The ratio of *Pina* to *Pinb* expression was generally lower in the hard non mutant genotypes. Hardness may be associated with differences in *Pin* expression and other factors and is not simply associated with mutations in the PIN protein coding sequences.

## Introduction

Grain hardness or endosperm texture, defined as having a hard endosperm (hard wheat/s) or soft endosperm (soft wheat/s), is one of the prime determinants of wheat quality as it has a major impact on the milling properties and end-use quality of the wheat. For commercial trading purposes wheat is mainly classified into soft, hard and very hard wheats based on grain hardness. As grain hardness is a fundamental attribute of wheat quality it has been studied for more than a century, and is reported to be mainly under genetic control with the environment having a negligible role [1–3]. Grain hardness is predominantly controlled by the *Puroindoline* (*Pin*) genes, *Pina* and *Pinb*, which are part of only the D sub-genome and located on chromosome 5 at the *Hardness* (*Ha)* locus. G*rain softness protein -1* (*GSP-1*), another gene tightly linked to the *Pin* genes on the *Ha* locus, was initially thought to be associated with grain hardness but later reports indicated otherwise [4–7]. During hexaploid wheat evolution, large genomic deletions in the short arm of chromosome 5 from the A and B sub-genomes caused the loss of the *Pin* genes but not the *GSP-1* gene in these sub-genomes of hexaploid wheat [8].

Wheat genotypes with wild type *Pina* (*Pina-D1a*) and *Pinb* (*Pinb-D1a*) alleles display soft kernel texture whereas mutation in any of the *Pin* genes results in a hard phenotype. Several mutant alleles of *Pina* and *Pinb* have been reported in the last two decades [9–11]. Among the mutant *Pin* alleles, *Pina-D1b* and *Pinb-D1b* are the most frequently observed mutant alleles in common wheat, worldwide [11]. *Pina-D1b* is a null allele caused by gene deletion, and *Pinb-D1b* is a result of a Gly46Ser change relative to the wild type *Pinb-D1a* [12]. Genotypes with the *Pina-D1b* allele display harder grain texture than those with *Pinb-D1b* [11]. Transgenic experiments have demonstrated the role of puroindoines in grain softening. In hard red spring wheat with a mutant *Pinb* allele (*Pina-D1a/**Pinb-D1b**)*, transformation with wild type *Pin* alleles has been shown to reduce grain hardness [13]. Similarly, transgenic expression of puroindoline in rice [14] and maize [15] has been shown to induce grain softness in these species which otherwise lack *Pin* genes.

Puroindoline protein isoforms, PINA and PINB, act together to form a friabilin protein [16] that binds to lipid molecules present on the starch surface through a hydrophobic tryptophan (trp) rich domain [17]. Friabilin prevents compact binding between starch and the protein matrix [18] which helps to soften the kernel texture. However, friabilin is less efficient in preventing this adhesion when composed of PIN proteins expressed by mutant *Pin* alleles resulting in grain hardness [19]. Mutation in *Pinb* has also been reported to reduce the amount of total PIN protein [20]. The Trp-rich domain in PINA contains 5 tryptophan residues whereas in PINB it contains 3 residues. Although PIN proteins act together their mode of action seems to be quite different most likely due to differences in their trp-rich domain. An *in vitro* study by Clifton, Sanders [21]shows that PINA forms self-assemblies in solution whereas PINB is dispersed in solution and PINB displays greater penetration into a lipid monolayer than PINA [17]. But, as the high resolution structures of these proteins is not yet known, the exact biochemical interactions between PIN proteins and starch remains unclear. However, it is believed that the trp-rich domain plays a key role in the biochemical action of the PIN proteins. According to *in vitro* studies this domain also provides antifungal and antibacterial properties to the PIN proteins [22].

The contribution of variations in *Pin* transcript abundance or patterns of *Pina* and *Pinb* expression to grain hardness remains unknown. Overexpression of wild type *Pin* alleles in hard wheat (*Pina-D1a/Pinb-D1b*) has been observed to reduce grain hardness [13]. In contrast, reduced expression of *Pin* genes through RNAi mediated silencing increases grain hardness [23]. *Pin* transcripts can be detected almost throughout the seed development period; from as early as 3 days post anthesis (DPA) until almost the end of maturity at 40 DPA. They are most abundant during the middle stages of seed development [13, 24–26]. *Pina* and *Pinb* have been reported to express at different levels (except *Pina* null) in some studies [12, 13, 27, 28], whereas, in other studies comparable levels (except *Pina* null) of gene expression has been reported [25, 28, 29]. This indicates the presence of different patterns of *Pina* and *Pinb* gene expression patterns in different genotypes. In this study we have studied these gene expression patterns in different genotypes.

In addition to *Pinb* on 5DS, several alleles of *Pinb-2* variants have also been discovered on chromosome 7 ABD [30–32]. *Pinb-2* variants share 57–60% homology with the *Pinb*. It has been suggested that they are likely to be involved in control of grain hardness [31]. However Giroux, Kim [33] showed that *Pinb-2* transcripts are expressed at very low levels compared to *Pina* and *Pinb* and proposed that they are less likely to influence grain hardness.

The molecular basis for the variation in grain hardness within a particular grain texture class and containing identical *Pin* alleles still remains unexplained. Identifying the role, if any, of other genes in controlling grain hardness will increase our understanding of variation in grain hardness. These genes may express differentially between soft and hard wheats. In this study, RNA-seq data generated by Next Generation Sequencing (NGS) was analysed to identify differentially expressed genes (DEGs) between hard and soft wheats. We also analysed gene expression of the *Pina*, *Pinb* and *GSP-1* ABD and *Pinb-2* ABD genes in thirty four wheat genotypes at two stages of seed development, 14 DPA and 30 DPA. The objective was to determine the patterns of *Pina*, *Pinb*, *GSP-1* ABD and *Pinb-2* ABD expression and their association with levels of grain hardness and to identify other candidate genes that might influence hardness.

## Materials and Methods

### Plant material

A selection of 33 different wheat genotypes from the global wheat gene pool were used for this experiment; Amurskaja 75, Arnhem, Banks, Batavia, Beyrouth 3, Bobwhite, Bowebird, D.E.S. 367, Dollarbird, EGA Gregory, Ellison, Gabo, Giza 139, Greece 25, Huandoy, India 211, India 259, India 37, Iraq 46, Jing Hoang No.1, Kite, Lerma Rojo, Martonvasari 13T, NW108A, NW25A, NW51A, NW93A, Pelada, Punjab 7, Qalbis, Saturno, Sunco, Tunis 24. Seeds were obtained from the Australian Winter Cereal Collection, Tamworth, Australia. Plants were grown in a growth room at day and night temperatures of 20 ^0^C and 18 ^0^C with 12 hours of light, at Southern Cross University. The whole caryopsis was collected at two developmental time points; 14 DPA and 30 DPA. Separate sets of plants were grown in a glasshouse as duplicates, for genotypes Banks, Ellison, Gabo, Gregory, Kite and Sunco.

### RNA isolation, cDNA preparation and NGS sequencing

Total RNA was isolated from the whole caryopsis using the Trizol reagent (Invitrogen, Carlsbad, USA) as published elsewhere [34]. To determine the total RNA concentration and quality, 2100 Bioanalyzer instrument (Agilent Technologies, Santa Clara, CA, USA) was used. cDNA was prepared and used to produce indexed Illumina NGS libraries which were then multiplexed to allow the sequencing of eight indexed libraries in one lane on a GA IIx Illumina sequencing platform. Seeds from the six duplicate genotypes grown in the glasshouse were also subjected to RNA isolation and NGS sequencing to provide replicate transcript profiles.

### Grain hardness measurement

Grain hardness index (HI) was measured using a single kernel characterisation system (SKCS 4100 crushing machine) according to AACC Method 55–31 (AACC, 1999). Tests were performed at BRI, Sydney. SKCS HI was obtained from measurement of 300 matured wheat grains.

### Quality analysis of sequence data

RNA sequencing of 33 genotypes generated a total of ~2.5 million to ~7.2 million reads varying with the genotype. All analysis of NGS data was undertaken using CLC Genomics Workbench V 8.0 (CLC Bio, Aarhus, Denmark). Illumina reads obtained from RNA-sequencing were trimmed for quality using default parameters to exclude calls with quality scores less than 20. Following trimming, all sequencing data was subjected to RNA Seq analysis using RNA-Seq tool.

### Identification of Pin alleles and RNA seq analysis

*Pina* and *Pinb* gene sequences were identified from the consensus sequence obtained from RNA-seq. Using the RNA-Seq analysis tool available in CLC workbench, gene expression was measured using RPKM normalization. Triticum aestivum gene index (TaGI) dataset generated by Dana-Farber Cancer Institute (DFCI) (ftp://occams.dfci.harvard.edu/pub/bio/tgi/data/Triticum_aestivum/) was used as a reference dataset for RNA-seq analysis. The parameters used for RNA–Seq analysis were, similarity fraction 0.9 and length fraction 0.8.

### Identification of Pin alleles in wheat genotypes

The RNA-seq analysis files of all of the wheat genotypes were used to extract full length coding sequences of the *Pina* and *Pinb* genes. However, in some genotypes part of the 5’-region of the coding sequence (up to 5 bp including the ATG start codon) of the *Pina* and/or the *Pinb* gene was missing. The sequence identity of the missing region of the *Pina* and the *Pinb* genes was determined, as outlined below, by PCR amplification of the pin genes followed by Sanger sequencing in both direction. Genomic DNA was extracted from the wheat genotypes NW93A, NW25A, NW108A, Amurskaja 75, Lermarojo and Banks using method developed by Furtado [34].Full length *Pina* was amplified using forward primer 5’ CATCTATTCATCTCCACCTGC 3’ and reverse primer 5’ GTGACAGTTTATTAGCTAGTC 3’ [11], and full length *Pinb* was amplified using forward primer 5’ GAGCCTCAACCCATCTATTCATC 3’ and reverse primer 5’ CAAGGGTGATTTTATTCATAG 3’ [11]. Using a thermal cycler (Biorad T100), the *Pin* genes were amplified by PCR, first for 10 cycles by denaturing at 94°C for 30 s, followed by annealing at 41°C for 30s, and extension at 72°C for 2 min, followed by 25 cycles by denaturing at 94°C for 30 s, followed by annealing at 45°C for 30s, and extension at 72°C for 2 min. Amplified bands for the *Pina* (524 bp) and the *Pinb* (595 bp) genes were first confirmed by agarose gel electrophoresis and then sequenced in both directions by Sanger Sequencing.

The full length coding sequences *Pina and Pinb* genes of all wheat genotypes were aligned to the corresponding soft *Pina* or *Pinb* gene allele to determine the presence or absence of mutant *Pin* alleles in the wheat genotypes.

### Identification of differentially expressed genes

The genotypes were divided into four groups based on grain hardness and presence of *Pin* mutant allele; Soft Non-Mutants, **SNM** (*PinaD1a/Pinb-D1a*; SKCS HI soft/ genetically soft), Hard Non-Mutants, **HNM** (*PinaD1a/Pinb-D1a*; SKCS HI hard/ genetically soft), Hard *Pina*-mutant, **HPAM** (*PinaD1b/Pinb-D1a*; SKCS HI hard/ genetically hard) and Hard *Pinb*-mutant, **HPBM** (*PinaD1a/Pinb-D1b*; SKCS HI hard/ genetically hard). These four groups were compared in nine different combinations to identify DEGs; SNM-HNM+HPAM+HPBM, SNM-HNM, SNM-HPAM, SNM-HPBM, HNM-HPAM, HNM-HPBM and non-mutants (Soft+HNM) vs HPAM+HPBM. ‘Empirical analysis of Differential Gene Expression’ test was performed to identify differentially expressed genes. Sequence ID’s with FDR p-value <1e-05 were selected as highly biologically significant differentially expressed sequences, candidate genes. These sequences were passed through NCBI BLASTx to find out similar protein sequences. BLAST hits were then functionally annotated using Blast2GO [35].

### Functional annotation through Blast2GO

BLAST hits were passed through array of different tools in Blast2GO; mapping, annotation, InterProScan, Merge InterProScan, annex and GO slim (plant). This gave the information about their biological process, molecular function and cellular component.

### Statistical analysis

Correlation between gene expression of *Pina*, *Pinb*, *GSP-1 ABD* and *Pinb-2 ABD*, and wheat grain hardness (SKCS HI) was evaluated based on linear and polynomial multiple regression analysis in RStudio software package. Data was grouped into 4 sets; *Pina-*mutant genotypes (*Pina-D1b*, *Pinb-D1a*), *Pinb-*mutant genotypes (*Pina-D1a*, *Pinb-D1b*), non-mutant genotypes (*Pina-D1a*, *Pinb-D1a*) and a last set included all the genotypes. A best fit model was selected by performing a stepwise Akaike information criterion (AIC) test in both directions mode followed by an Anova test.

## Results

### SKCS Hardness Index and Pin allele identification

Grain hardness as measured using a Single Kernel Characterisation System (SKCS) and the nature of the *Pin* alleles of the wheat genotypes included in this study are shown in Table 1. Genotypes were classified as soft wheat or hard wheat if their SKCS hardness index (SKCS HI) was below or above 50, respectively [36–38]. Using the full-length coding sequence of the *Pina* and *Pinb* genes obtained from RNA-Seq analysis or by PCR amplification, wheat genotypes were identified as consisting of no mutations in the *Pina* and *Pinb* genes (*Pina-D1a/Pinb-D1a*) or consisting of mutations in either one of the *Pin* genes but not both (*Pina-D1a/Pinb-D1b or Pina-D1b/Pinb-D1a*) (Table 1). Based on the absence or presence of mutant *Pin* alleles, the wheat genotypes were divided into three main groups; a non-mutant group (*Pina-D1a/Pinb-D1a*) of seventeen genotypes comprising both soft and hard (hard non-mutant, HNM) (genetically soft) wheats, a hard *Pina*-mutant (HPAM) group (*Pina-D1b/Pinb-D1a*) of nine hard genotypes, and a hard *Pinb*-mutant group (HPBM) (*Pina-D1a/Pinb-D1b*) of seven hard genotypes (Table 1). Within the non-mutant group, the genotypes; India 259, Greece 25, Beyrouth 3, Saturno, Amurskaja 75 and four genotypes from Nepal (NW108A, NW93A, NW25A and NW51A) were found to be hard (HNM) wheats even though these nine genotypes had no mutations in the *Pin* genes.

**Table 1:** Grain hardness Index (HI) based on presence of Pin alleles genotypes were grouped into three different groups. SD—Standard deviation.

Genotype	AUS Code	Country of origin	SKCS HI	SD +	Classified as S, soft; H, hard wheat	*Pin* alleles	Group	
Huandoy	14423	PER	39	16	S	*Pina-D1a (wild type) & Pinb-D1a (wild type)*		Non- mutant group	
Lerma Rojo	473	MEX	42	19	S				
D.E.S. 367	4385	GRC	42	15	S				
Tunis 24	13160	TUN	43	16	S				
Giza 139	12957	EGY	44	14	S				
Pelada	7449	VEN	44	23	S				
Qalbis	33372	AUS	49	14	S				
India 211	15330	IND	50	12	S				
NW108A	15036	NPL	53	19	H				
India 259	4838	IND	57	16	H				
Greece 25	4606	GRC	57	14	H				
Saturno	24431	MEX	60	25	H				
Beyrouth 3	4205	LBN	61	12	H				
Amurskaja 75	20438	SUN	66	23	H				
NW93A	15022	NPL	74	17	H				
NW51A	14996	NPL	74	16	H				
NW25A	14981	NPL	83	17	H				
Jing Hoang No. 1	17863	CHN	53	20	H	*Pina-D1a* (wild type) & *Pinb-D1b* (mutant)		Pinb-mutant group	
Batavia	25271	AUS	71	15	H				
EGA Gregory	34283	AUS	71	15	H				
Sunco	23455	AUS	78	16	H				
Martonvasari 13T	24341	HUN	82	17	H				
Banks	20599	AUS	87	16	H				
Kite	16035	AUS	92	16	H				
Ellison	33371	AUS	82	15	H	*Pina-D1b* (mutant) & *Pinb-D1a* (wild-type)		Pina-mutant group	
Arnhem	25607	AUS	83	16	H				
India 37	4671	IND	87	13	H				
Dollarbird	23824	AUS	87	13	H				
Punjab 7	879	IND	88	13	H				
Gabo	246	AUS	93	15	H				
Iraq 46	28823	IRAQ	94	15	H				
Bowerbird	30434	AUS	96	16	H				
Bobwhite S-26	30252	MEX	107	17	H				

Wheat Seeds were sourced from the Australian Winter Cereal Collection (AWCC), *Tamworth*, Australia.

### Differentially expressed genes in hard wheats compared to soft wheats

Gene expression measurements were found to be comparable between replicate experiments for the same genotype (S3 Table). RNA-Seq analysis of expression data of soft wheats and of all hard wheats together i.e. Soft vs HNM+HPAM+HPBM, identified three genes differentially expressed at a FDR corrected value of p<1e-05 (Table 2A). However, the distribution of gene expression values as RPKM, for these three genes, across the two groups of wheat genotypes was similar except for one outlier, implying that these genes may not be significantly associated with grain hardness.

**Table 2:** Differentially expressed genes identified in the hard wheats when compared to the soft wheats. 2a. Soft vs HNM+HPAM+HPBM, 2b. Soft vs HPAM, 2c. Soft vs HPBM, 2d Soft vs HNM.

TAGI seq. ID	Fold change (RPKM)	FDR p-value correction	Sequence Description
**2a. Soft vs HNM+HPAM+HPBM** Differentially expressed genes in HNM+HPAM+HPBM group when compared to Soft group.			
TC393450	-15.99	2.52E-07	high molecular weight glutenin subunit
TC388873	-46.47	1.66E-06	60s ribosomal protein l23
CV066856	-35.82	3.23E-05	---NA---
**2b. Soft VS HPAM** Differentially expressed genes in HPAM group when compared to Soft group.			
TC423373	-1,768.05	3.60E-102	puroindoline-a
TC434025	-82.76	3.04E-15	alpha- partial
TC393944	-469.84	3.59E-15	alpha- partial
TC401139	-100.66	1.16E-08	calreticulin interacted protein
NP9350187	248.04	1.72E-07	low molecular weight glutenin
BE413821	124.96	2.33E-06	alpha- partial
TC448640	-135.24	3.42E-06	---NA---
TC448029	-49.14	7.01E-06	proteasome subunit beta type-2-like
BJ221811	11.73	8.92E-06	disease resistance protein rpm1
BE398961	-76.85	1.97E-06	---NA---
**2c. Soft VS HPBM** Differentially expressed genes in HPBM group when compared to Soft group.			
CA602991	124.46	7.16E-10	inactive poly
TC400917	-100.24	8.05E-07	histone h4
TC372980	-27.52	2.13E-06	40s ribosomal protein s12
TC420043	3608.9	2.56E-05	hypothetical protein TRIUR3_06809
**2d. Soft VS HNM** Differentially expressed genes in HNM group when compared to Soft group.			
TC420856	98.71	9.31E-14	---NA---
TC372034	71.66	5.22E-13	eh domain-containing protein 1-like
CA636509	58.21	1.50E-06	glycosyltransferase
TC427661	20.08	1.80E-06	---NA---

TAGI sequence ID = Triticum Aestivum Gene Indices sequence ID; RPKM = reads per kilobase per million; NA = not available; FDR = false discovery rate; HNM = hard non-mutant; HPAM = hard *Pina*-mutant; HPBM = hard *Pinb*-mutant.

### Differentially expressed genes in hard Pina mutant (HPAM) wheats compared to soft wheats

When the HPAM group was compared with the soft group, 10 differentially expressed genes (DEGs) were identified (Table 2B). As expected, *Pina* was the most highly differentially expressed gene. The significance value and fold change value of no other gene was even close to that of *Pina*. In addition, none of the 10 DEGs identified in this analysis were common with DEGs identified in comparison of the soft group with the HNM+HPAM+HPBM group.

### Differentially expressed genes in hard Pinb mutant wheats (HPBM) compared to soft wheats

When the HPBM group was compared with the soft group, DEGs were identified (Table 2C). *Pinb* (*Pinb-D1b*) was not identified as a DEG even up to a significance value at FDR corrected p-value of 0.01. The DEGs identified in this analysis were different to those identified in the soft vs HNM+HPAM+HPBM and the soft vs HPAM groups.

### Differentially expressed in hard non-mutants (HNM) compared to soft, HPAM and HPBM groups

In the HNM group, grain hardness was observed but without mutation in either the *Pina* or the *Pinb* gene. To identify genes with expression contributing to grain hardness in the absence of the *Pina/b* mutation we compared the HNM group with other non-mutant wheats which are soft; soft vs HNM. In this comparison 4 DEGs were identified (Table 2D) which were different to the DEGs observed in the three earlier comparisons. *Pin* genes were not among the differentially expressed genes even at a FDR p-value up to 0.01.

The HNM group was also compared separately with the HPAM and the HPBM group, where 180 (173 up-regulated in HNM), 26 (23 up-regulated in HNM) DEGs were identified, respectively (S1 Table and Table 3). Among these two sets of DEGs, 9 DEGs were common and were up-regulated in HNM group (S1 Table). HNM vs HPAM and HNM vs HPBM comparisons had no DEGs in common with the SNM vs HNM comparison.

**Table 3:** Top down-regulated and up-regulated differentially expressed genes identified in HPAM (3a) and HPBM (3b) when compared to HNM group.

TAGI seq. ID	Fold change (RPKM)	FDR p-value correction	Sequence Description
**3a. HNM vs HPAM** Differentially expressed genes in HPAM group when compared to HNM group.			
TC423373	-1310.78	1.25E-43	puroindoline-a
CD915505	902.62	9.63E-19	low-molecular-weight glutenin subunit
NP9350187	166.51	5.41E-07	low molecular weight glutenin
CJ520220	-843.82	6.98E-07	---NA---
BE413821	117.62	1.07E-06	alpha- partial
TC393944	-526.26	1.14E-06	alpha- partial
TC458604	-193.09	2.45E-06	---NA---
TC393980	40.28	2.46E-06	low-molecular-weight glutenin subunit
TC391918	28.3	6.57E-06	formate dehydrogenase mitochondrial-like
TC412255	-152.77	9.59E-06	---NA---
**3b. HNM vs HPBM** Differentially expressed genes in HPBM group when compared to HNM group.			
CK194194	-78.27	3.13E-17	fructose-bisphosphate aldolase
CA602991	85.03	6.94E-08	inactive poly
TC450096	-66.92	8.68E-07	---NA---
TC375635	11.6	1.91E-06	dihydrolipoamide s-acetyltransferase
CK162779	-233.06	2.66E-06	lrr receptor-like serine threonine-protein kinase fls2
CD897082	-62.56	4.13E-06	---NA---
TC457676	-180.99	4.15E-06	---NA---
TC420456	6.94	6.2E-06	ac078948_18 serine protease

TAGI sequence ID = Triticum Aestivum Gene Indices sequence ID; RPKM = reads per kilobase per million; NA = not available; FDR = false discovery rate; HNM = hard non-mutant; HPAM = hard *Pina*-mutant; HPBM = hard *Pinb*-mutant.

### Differentially expressed genes in Pin mutant wheats (HPAM & HPBM) compared to non-mutants (soft & HNM)

When all the Non-mutant (soft and HNM) wheats were compared to HPAM and HPBM groups together, 345 differentially expressed genes were identified (S2 Table) and of these 337 DEGs were up-regulated in non-mutants. Comparison of these 345 DEGs with DEGs from soft VS HPAM, soft vs HPBM, HNM vs HPAM and HNM vs HPBM comparisons identified 2, 3, 128 and 15 common genes, respectively.

### Differential expression of *Pina*, *Pinb* and *GSP-1* ABD and *Pinb*-2 ABD at two stages of seed development

**Fig 1:**
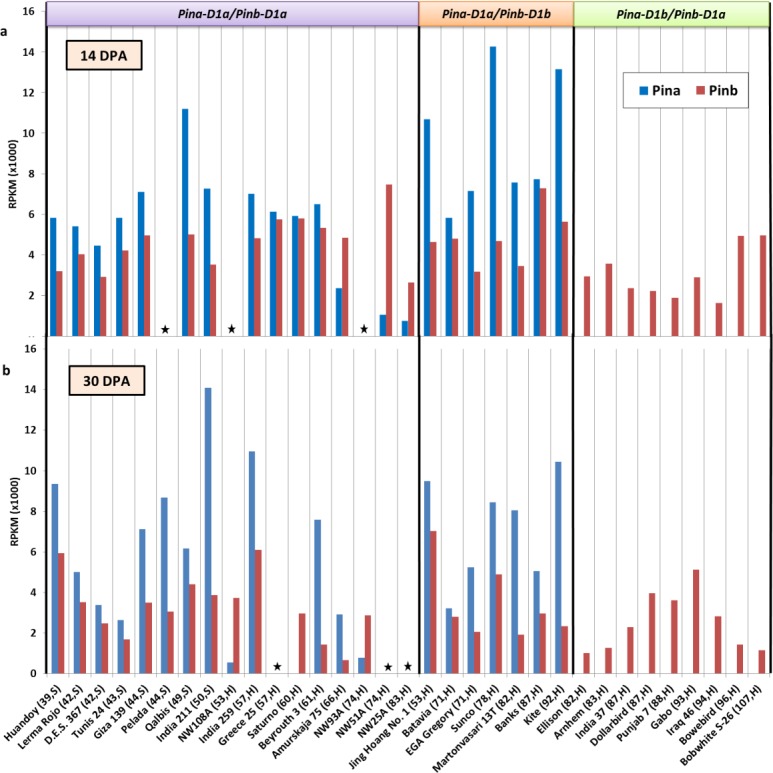
Expression of the *Pina* and *Pinb* genes in developing seeds of several wheat genotypes. a,b gene expression data at 14 and 30 days post anthesis (dpa), respectively; RPKM, reads per kilo base per million mapped reads. Details in brackets after genotype names on the X-axis indicates grain hardness index and endosperm texture of genotypes classified as hard (H) or soft (S) wheats. Genotypes with HI above 50 were classified as Hard. Wheat genotypes in each group are arranged left to right with respect to increasing hardness index. Boxes on the top indicate *Pin* alleles present in the genotypes. cDNA prepared from RNA extracted from developing wheat seeds at 14 DPA, was subjected to next generation sequencing. RNA-seq analysis was undertaken using CLC Genomic Workbench V8 to determine gene expression of *Pina* and *Pinb*. The star symbol indicates data not available.

**Fig 2:**
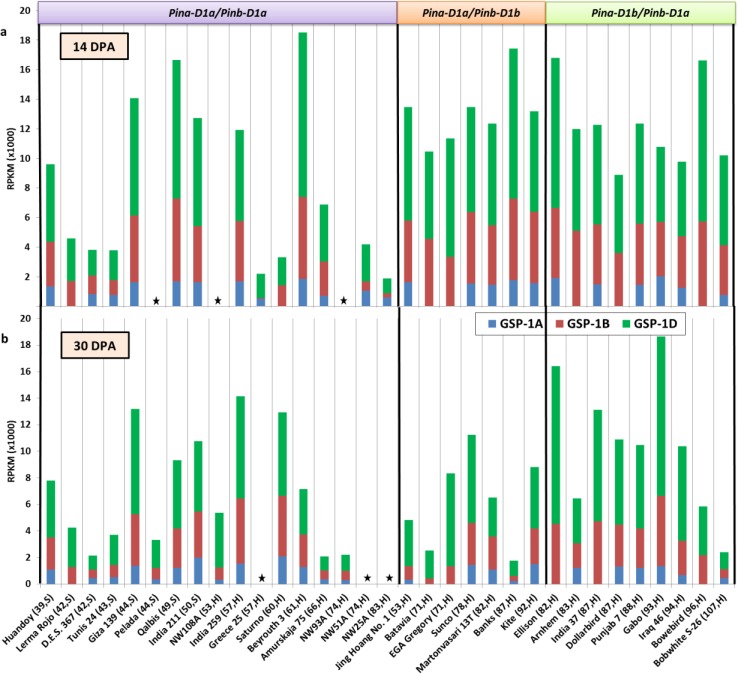
Expression of the *GSP-1*A, -1B, -1D genes in developing seeds of several wheat genotypes. a, b gene expression data at 14 and 30 days post anthesis (DPA) respectively; RPKM, reads per kilo base per million mapped reads. Boxes on the top indicate *Pin* alleles present in the genotypes. Details in brackets after genotype names on the X-axis indicates grain hardness index and endosperm texture of genotypes classified as hard (H) or soft (S) wheats. The asterisk indicates data not available.

**Fig 3:**
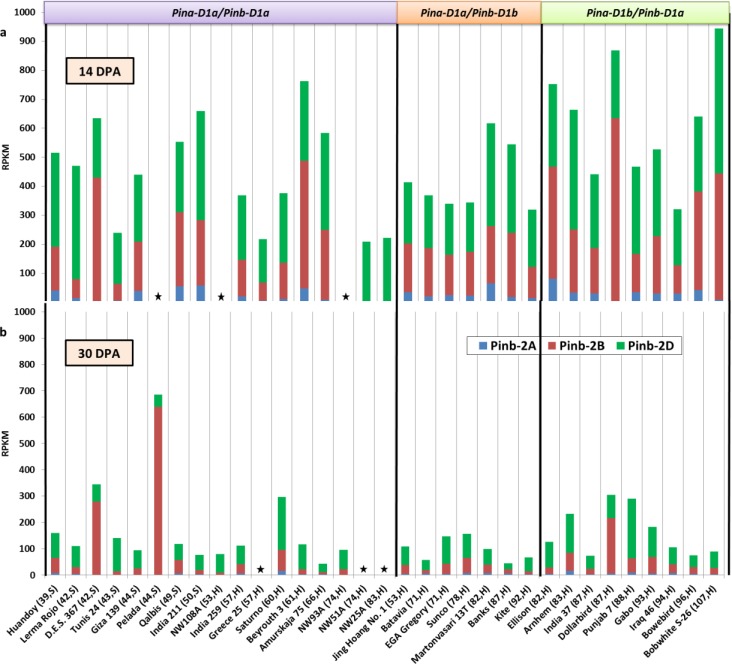
Expression of the *Pinb-2*A, -2B, -2D genes in developing seeds of several wheat genotypes. a, b gene expression data at 14 and 30 days post anthesis (dpa) respectively; RPKM, reads per kilo base per million mapped reads. Boxes on the top indicate *Pin* alleles present in the genotypes. Details in brackets after genotype names on the X-axis indicates grain hardness index and endosperm texture of genotypes classified as hard (H) or soft (S) wheats. The asterisk indicates data not available.

Differential expression of *Pina*, *Pinb*, *GSP-1A*, *-1B*, *-1D* and *Pinb-2A*, *-2B*, *-2D* for all genotypes at 14 DPA and 30 DPA is shown in Figs 1–3. The pattern but not the proportion of differential gene expression of *Pina*, *Pinb*, *GSP-1 ABD* and *Pinb-2 ABD* was roughly the same at both time points for most genotypes.

In the non-mutant group, *Pina (Pina-D1a)* expression was found to be generally higher than *Pinb* expression (Fig 1). However, within the non-mutant group a specific pattern of *Pin* gene expression was observed at 14 DPA or 30 DPA in the hard wheat genotypes Amurskaja 75, NW25A, NW51A, NW93A and NW108A. The pattern of *Pin* gene expression when compared to the other genotypes in the non-mutant group reflected the reduced levels of *Pin*a expression and in addition the increased level of *Pinb* expression when compared to *Pina* expression. Genotypes with increasing hardness in this group did not show an observable reduction in *Pinb* expression. However, reduced *Pina* expression was observed in those genotypes, as outlined above, with the highest hardness index in this group.

In the *Pinb*-mutant group, *Pina (Pina-D1a)* expression was found to be higher than *Pinb* expression (Fig 1). Genotypes with increasing hardness in this group did not show an observable reduction in the expression levels of *Pina* or *Pinb-D1b*.

In the *Pina*-mutant group, expression of *Pina* was not detected as expected, but the expression of *Pinb* was lower compared to the non-mutant and the *Pinb*-mutant group. Genotypes with increased hardness did not show an observable correlation with *Pinb* expression levels (Fig 1).

In case of *GSP-1A*, *-1B*, *-1D* and *Pinb-2A*, *-2B*, *-2D*, the highest expression was contributed by the D sub-genome allele followed by the B sub-genome allele and then the A sub-genome allele (Figs 2 and 3).

### Total gene expression of Pina, Pinb and GSP-1 ABD and Pinb-2 ABD at two stages of seed development

**Fig 4:**
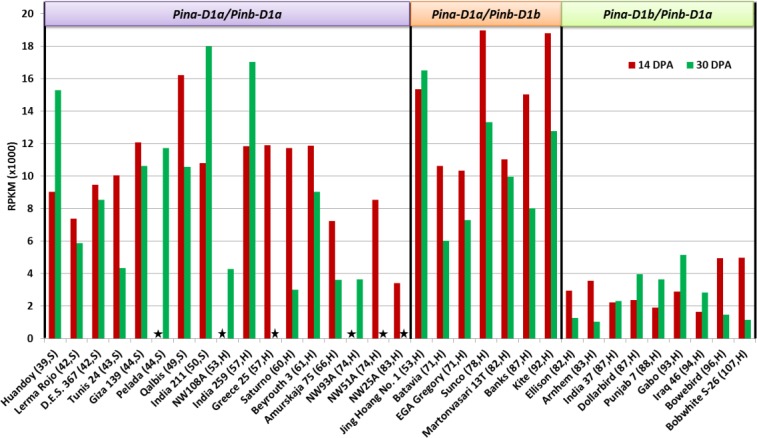
Total expression of *Pin* genes (*Pina* + *Pinb*) in developing wheat seeds of several wheat genotypes at 14- and 30-days post anthesis. Details in brackets after genotype names on the X-axis indicates grain hardness index and endosperm texture of genotypes classified as hard (H) or soft (S) wheats. Grain hardness was measured by SKCS analysis and genotypes with HI above 50 were labelled as hard. Details in brackets after genotype names on the X-axis indicates grain hardness index and endosperm texture of genotypes classified as hard (H) or soft (S) wheats. Boxes on the top indicate *Pin* alleles present in the genotypes. Transcriptome analysis by next generation sequencing data was undertaken to determine total *Pin* gene expression which was then normalised and expressed as reads per kilo base per million mapped reads (RPKM). The asterisk indicates data not available.

Total *Pin* gene expression in developing seeds at 14 and 30 DPA (Fig 4) was generally higher in most genotypes at 14 DPA than at 30 DPA. The total *Pin* gene expression, within each of the *Pin*-groups, showed no observable correlation with increasing SKCS-HI or with genotypes found to be soft or hard. In the *Pina*-mutant group, total *Pin* gene expression was much lower when compared to the non-mutant group or the *Pinb*-mutant group.

Total *GSP-1* expression was also observed to be slightly reduced at 30 DPA (Fig 2). Total *Pinb-2* expression at 30 DPA was substantially reduced as compared to 14 DPA (Fig 3). Analysis showed a higher expression of *GSP-1* in *Pina-* and *Pinb-* mutant genotypes than in non-mutant genotypes but no such pattern was observed for *Pinb-2*.

### Statistical analysis of associations with hardness

The three groups of wheat genotypes, non-mutants (Soft and HNM), HPAM and HPBM, were analysed separately and together to find out which of the *Pin*, *Pinb-2* or the *GSP-1* genes had the greatest impact on SKCS HI. In a polynomial regression model which included all the genotypes and the individual genes, *Pina*, *Pinb*, *GSP-1 ABD* and *Pinb-2 ABD*, our data suggests that the expression of *Pina* + *Pinb* + *GSP-1B + Pinb-2D* explained 62% of the variation at 14 DPA with a p-value of 0.0001 (Table 4). The correlation between *Pina* gene expression and SKCS HI was the highest among all genes in a group containing all genotypes (Table 4). The combined or individual effects of *GSP-1* ABD and *Pinb*-2 ABD alleles did not show significant correlation with SKCS HI in any group. Within the non-mutant group, an interactive effect of *Pina* x *Pinb* explained 80% (p-value 0.01) of the variation among SKCS HI.

**Table 4:** Association between *Pina*, *Pinb* and *GSP-1 ABD and Pinb-2 ABD* gene expression measurements and SKCS hardness index, at 14 DPA. No significant association was observed at 30 DPA. Genotypes were grouped into four groups for statistical analysis; all genotypes, non-mutants, Pina-mutants and Pinb-mutants. Additive and interactive effects of gene expression were analysed using linear and polynomial regression model. Only significant associations are listed.

Group	Genes	R^2^ value	P value
Allgenotypes	*Pina+Pinb+GSP-1A+Pinb-2A* ***#***	0.39	0.0007
	*Pina+Pinb+GSP-1B+Pinb-2D* *	0.62	0.0001
	*Pina +Pinb* *	0.56	2.562e-05
	*Pina* *	0.53	7.808e-06
	*Pina X Pinb* *	0.57	0.00038
Non-mutant	*Pina X Pinb* ***#***	0.60	0.005
	*Pina + Pinb* *	0.57	0.01
	*Pina X Pinb* *	0.81	0.01
	*Pina* *	0.53	0.005

**#** Linear regression

* Polynomial regression, + Additive effect of gene expression, X Interactive effect of gene expression

## Discussion

In this study we have addressed two questions; firstly, which genes are differentially expressed in hard wheats compared to soft wheats and secondly, does differential expression of *Pina* and *Pinb* genes explain variation in wheat grain hardness.

The TAGI reference dataset was used for RNA-seq analysis in this study as it contains more genes compared to the IWGSC reference dataset. Recently, a gene responsible for good bread making, *wheat bread making* gene, was discovered using the TAGI database[39]. This gene is not present in the IWGSC database. The IWGSC survey sequence database is in the process of completion and is updated frequently by addition of new genes. The majority of grain hardness is explained by puroindolines, however, some variation still remains unexplained possibly due to the involvement of other genes playing a minor role in the control of grain hardness. A number of QTLs have been shown to be associated with grain hardness, on chromosome 4A, 4B [40], 1A, 2B, 2D, 3B, 7A, 7B [41], 6A [42], 1B [43]. However, no genes other than the *Pin* genes have been associated with grain hardness. We hypothesised that genes other than *Pin* genes that control grain hardness may be expressed differentially between the soft and the hard wheats. We identified these DEGs by analysing the transcriptome of several soft and hard wheat genotypes. Four groups of wheats; soft, HNM, HPAM and HPBM were compared with each other in different combinations to identify differentially expressed genes. This provided the group specific and the common DEGs within different groups.

The most significant differential expression of *Pina* in comparisons between soft vs HPAM and HNM vs HPAM supports the vital role of *Pina* in controlling grain hardness. However, *Pina* or *Pinb* was not identified as a differentially expressed gene in comparison of the soft vs HNM group. This indicates that although grain hardness is greatly influenced by mutation in the *Pin* genes, other factors may also be involved in control of this trait. In the genes which were differentially expressed between soft+HNM group and HPAM+HPBM group it was observed that most of the genes were down-regulated in hard wheats (HPAM+HPBM). This down-regulation of gene expression could be the influence of grain hardness or it may contribute to the grain hardness. No annotation was available for some of the significant DEGs. None of the other annotated DEGs identified in different comparisons has been previously reported to be associated with wheat grain hardness. Further study is required to determine the role of candidate genes identified in this study. Up-regulation of glutenins and gliadins (Table 2 and S1 Table) in the hard wheats is most likely a mere coincidence as the hard wheats have been selected for higher gluten content for bread-making. The differential expression of these genes may be because of human selection for different wheat quality traits in hard and soft wheats rather than their association with the determination of hardness.

*Pinb-2* variants and *GSP-1* have been suggested as possible candidates for control of grain hardness [7, 31]. Results of this study indicate that the *GSP-1* and the *Pinb-2* do not have a significant influence on grain hardness on their own as has been reported in other studies [7, 19, 33]. However, the possibility of a combined influence of the *Pinb-2*, *GSP-1* and *Pin* genes in control of grain hardness is likely as the expression of *Pina+Pinb+GSP-1B+Pinb-2D* explained 62% of the variation in grain hardness whereas *Pina*+*Pinb* explained 56% across all genotypes in this study.

*Pina* and the *Pinb* still remain the only major genes to influence grain hardness. In the non-mutant group, the additive effect of *Pina*+*Pinb* expression explained 60% of the variation in grain hardness whereas the interactive effect explained 80%. However, the individual effect of *Pina* expression was the highest (53%) (Table 4). Higher expression of *Pina* rather than *Pinb* was one of the most consistent patterns of gene expression observed in these genotypes, except for the case of the *Pina*-mutants (Fig 1). This observation has been reported in earlier studies [12, 13, 27, 28]. At the protein level, a higher amount of PINA has been suggested to be associated with increased starch bound PIN, which is supposedly associated with increased grain softness [44]. We found some wheat genotypes with a hard grain texture without the presence of any mutant-*Pina* or -*Pinb* alleles. This is a very significant observation which provides an exception to the widely accepted view that wheats with wild-type *Pin* alleles display soft grain texture but a mutation in any of the *Pin* alleles leads to hard grain texture. Among the nine genotypes with hard grain texture within the non-mutant group, five genotypes showed a distinct pattern of the *Pina* and the *Pinb* gene expression at either 14 or 30 DPA (Fig 4). Generally, the *Pina* transcripts are expressed at a higher level than the *Pinb* but in these genotypes we found the opposite, with the *Pinb* expression levels exceeding the *Pina* expression levels. This result provides strong evidence that reduced expression of *Pina* and *Pinb* alleles, and not just protein structure, plays a critical role in determining grain hardness. In agreement with these results Swan, Meyer [45] reported that grain softness is explained better by the proportional amounts of the PINA and the PINB proteins rather than the total PIN content [45]. Hard grain texture in genotypes containing wild type *Pina* and *Pinb* has been reported earlier in Australian cultivars Cook and Diaz, with a PINA:PINB ratio of about 2:1 (ELISA test) [27]. However, gene expression wasn’t examined in that study.

A slightly lower *Pinb* expression was observed in *Pina*-mutants (HPAM) compared to other groups (Fig 1). Likewise, a lower amount of the PINB protein has been reported in wheat genotypes with a *Pina* null mutation [27]. Amoroso, Longobardo [28] suggested that PINB is expressed in *Pina* null genotypes but is later degraded due to the absence of the PINA which was suggested to stabilise PINB. In contrast, Wanjugi, Hogg [46] reported that PINA and PINB can act independently and produce intermediate grain texture but are more effective in producing soft grains when acting together. A transgenic study by Hogg, Sripo [13] showed that hard red spring wheat (Hi-line) with a mutant *Pinb* allele (*Pina-D1a/Pinb-D1b*), when transformed with wild type *Pin*-a or–b alleles produces soft grains. However, lines transformed with *Pinb-D1a* were softer than the lines transformed with *Pina-D1a* and in addition there was no correlation with levels of PIN expression and hardness. This demonstrates that excessive expression of one wild type *Pin* allele does not compensate for another mutant *Pin* allele and also indicated that PINA and PINB act together. Comparable levels of *Pina* and *Pinb* expression in some non-mutants and *Pinb*-mutants suggests that mutation in the *Pin-b* gene does not necessarily alter the expression levels of *Pin* genes. A study by Gasparis, Orczyk [23] using RNAi mediated silencing of *Pin* genes also reported that puroindoline transcript abundance or protein content is not altered by mutation. However, mutation may interfere with PIN protein functionality and stability. Greater association of *Pin* transcripts at 14 DPA with grain hardness is most likely due to the abundance of transcripts whereas less association at 30 DPA is most likely due to reduced gene expression. The total gene expression process starts to slow down as the seed proceeds towards maturity [47].

Study of 5’ and the 3’ sequences of the *Pin* genes may explain differences in expression levels of *Pin* genes. Mutations in these regions are likely to affect gene expression levels. Further understanding of grain hardness may allow selection of specific levels of gene expression to breed for specific level of grain hardness and not just for broader classes of hardness. A more complete understanding of the regulatory sequences at these loci may allow a more complete explanation of genetic variation in hardness in wheat and allow reliable selection for hardness based upon sequence specific markers.

## Conclusions

Results of this study show that grain hardness in some wheat genotypes containing wild type *Pin* alleles is explained by higher expression of *Pinb* than *Pina*. Puroindoline genes still remain the major determinants of grain hardness. Expression of *Pin* genes at earlier stages of grain development determines grain hardness. Variation in grain hardness (SKCS HI) among the different genotypes within a particular class of hardness couldn’t be explained by patterns of *Pina* and *Pinb* expression. Several statistically significant differentially expressed genes were identified between soft wheats and hard wheats. Further investigation of these genes may provide more understanding of wheat grain hardness.

## Supporting Information

S1 Table Differentiallyexpressed genes identified between the HNM group and the HPAM group (1a), the HPBM group (1b) and common DEGs (1c).
(DOC)

S2 Table Differentially expressed genes identified between the Pina-mutatnts (HPAM+HPBM) and the non-mutant group (soft+HNM). (DOC)

S3 Table *Pin* gene expression results for duplicate experiments for the same genotypes at 14 DPA. Duplicates were grown in a glasshouse and analysed to exmine reprodctibility of expression patterns.
(DOC)
