# Supplementary material for: Influence of Gene Expression on Hardness in Wheat
Source: PLoS One. 2016 Oct 14;11(10):e0164746. doi: 10.1371/journal.pone.0164746 (PMC5065149; doi:10.1371/journal.pone.0164746)
Supplement: S1 Table — Differentiallyexpressed genes identified between the HNM group and the HPAM group (1a), the HPBM group (1b) and common DEGs (1c). (DOC) [file pone.0164746.s001.doc]

**S1 Table.** Differentially expressed genes identified between the HNM group and the HPAM group (**1a**), the HPBM group (1**b**), and common DEGs (**1c**).

| **Feature ID** | **Fold change** | **FDR p-value correction** | **Seq. Description** | | **Seq. Length** | **#GOs** | **GOs** | | **InterProScan** |
| --- | --- | --- | --- | --- | --- | --- | --- | --- | --- |
| **1a. HNM vs HPAM** Differentially expressed genes identified in HPAM group when compared to HNM group. | | | | | | | | | |
| TC423373 | -1310.78 | 1.25E-43 | puroindoline-a | | 761 | 5 | C:extracellular space; P:response to external stimulus; P:response to stress; P:response to biotic stimulus; C:membrane | | IPR001376 (PRINTS); SIGNAL_PEPTIDE (PHOBIUS); SIGNAL_PEPTIDE_C_REGION (PHOBIUS); NON_CYTOPLASMIC_DOMAIN (PHOBIUS); SIGNAL_PEPTIDE_N_REGION (PHOBIUS); SIGNAL_PEPTIDE_H_REGION (PHOBIUS); SignalP-noTM (SIGNALP_GRAM_NEGATIVE); SignalP-TM (SIGNALP_GRAM_POSITIVE); SignalP-noTM (SIGNALP_EUK) |
| CJ520220 | -843.82 | 6.98E-07 | ---NA--- | | 336 | 0 | - | | no IPS match |
| TC393944 | -526.26 | 1.14E-06 | alpha- partial | | 898 | 7 | C:cytoplasm; F:structural molecule activity; F:nucleotide binding; P:cellular process; P:cellular component organization; F:hydrolase activity; C:cytoskeleton | | no IPS match |
| TC458604 | -193.09 | 2.45E-06 | ---NA--- | | 506 | 0 | - | | no IPS match |
| TC412255 | -152.77 | 9.59E-06 | ---NA--- | | 543 | 0 | - | | PTHR31673:SF3 (PANTHER); PTHR31673 (PANTHER); TRANSMEMBRANE (PHOBIUS); CYTOPLASMIC_DOMAIN (PHOBIUS); NON_CYTOPLASMIC_DOMAIN (PHOBIUS); TMhelix (TMHMM) |
| CA680050 | -151.65 | 8.51E-07 | ---NA--- | | 636 | 0 | - | | no IPS match |
| TC448640 | -140.19 | 7.53E-06 | ---NA--- | | 268 | 0 | - | | Coil (COILS); TRANSMEMBRANE (PHOBIUS); NON_CYTOPLASMIC_DOMAIN (PHOBIUS); CYTOPLASMIC_DOMAIN (PHOBIUS); TMhelix (TMHMM) |
| TC434025 | -134.5 | 3.76E-14 | alpha- partial | | 875 | 11 | F:GTP binding; C:protein complex; P:protein polymerization; F:structural constituent of cytoskeleton; F:GTPase activity; P:obsolete GTP catabolic process; P:microtubule-based process; C:microtubule; F:nucleotide binding; C:cytoplasm; C:cytoskeleton | | IPR002411 (PRINTS); IPR006106 (PRINTS); IPR016140 (SMART); IPR013771 (G3DSA:1.10.120.GENE3D); IPR016140 (PFAM); IPR016309 (PIRSF); IPR006105 (PROSITE_PATTERNS); SIGNAL_PEPTIDE_N_REGION (PHOBIUS); SIGNAL_PEPTIDE_H_REGION (PHOBIUS); SIGNAL_PEPTIDE_C_REGION (PHOBIUS); NON_CYTOPLASMIC_DOMAIN (PHOBIUS); SIGNAL_PEPTIDE (PHOBIUS); SignalP-noTM (SIGNALP_GRAM_NEGATIVE); SignalP-TM (SIGNALP_GRAM_POSITIVE); SignalP-noTM (SIGNALP_EUK); IPR016140 (SUPERFAMILY); TMhelix (TMHMM) |
| TC401729 | -110.91 | 9.89E-07 | rrna intron-encoded homing endonuclease | | 1066 | 0 | - | | IPR000425 (PRINTS); IPR023271 (G3DSA:1.20.1080.GENE3D); IPR000425 (PFAM); IPR000425 (PANTHER); PTHR19139:SF30 (PANTHER); NON_CYTOPLASMIC_DOMAIN (PHOBIUS); TRANSMEMBRANE (PHOBIUS); TRANSMEMBRANE (PHOBIUS); CYTOPLASMIC_DOMAIN (PHOBIUS); CYTOPLASMIC_DOMAIN (PHOBIUS); NON_CYTOPLASMIC_DOMAIN (PHOBIUS); TRANSMEMBRANE (PHOBIUS); IPR023271 (SUPERFAMILY); TMhelix (TMHMM); TMhelix (TMHMM); TMhelix (TMHMM) |
| CJ726075 | -96.71 | 5.09E-07 | hypothetical protein TRIUR3_07013 | | 761 | 0 | - | | IPR008547 (PFAM); PTHR12265:SF3 (PANTHER); IPR008547 (PANTHER) |
| CK162779 | -96.28 | 1.58E-06 | lrr receptor-like serine threonine-protein kinase fls2 | | 1025 | 9 | C:cytoplasm; F:kinase activity; P:cellular protein modification process; F:nucleotide binding; C:membrane; C:plastid; F:protein binding; P:metabolic process; P:cellular process | | no IPS match |
| TC445483 | -93.67 | 1.14E-06 | ---NA--- | | 314 | 0 | - | | no IPS match |
| BE398961 | -92.86 | 8.31E-11 | ---NA--- | | 266 | 0 | - | | no IPS match |
| TC440571 | -92.53 | 3.27E-07 | ---NA--- | | 676 | 0 | - | | IPR000357 (PFAM); IPR011989 (G3DSA:1.25.10.GENE3D); PTHR10648 (PANTHER); IPR021133 (PROSITE_PROFILES); IPR021133 (PROSITE_PROFILES); IPR016024 (SUPERFAMILY) |
| TC449482 | -90.54 | 1.79E-06 | cytochrome p450 like_tbp | | 1124 | 1 | F:catalytic activity | | no IPS match |
| TC456067 | -90.13 | 4.74E-06 | histone h4-like | | 476 | 27 | P:cell cycle; C:membrane; C:vacuole; C:nucleoplasm; F:catalytic activity; P:regulation of gene expression, epigenetic; P:nucleobase-containing compound metabolic process; P:biosynthetic process; F:RNA binding; C:plastid; P:cellular component organization; P:DNA metabolic process; C:intracellular; C:nucleolus; C:cytosol; F:protein binding; P:cell differentiation; P:multicellular organismal development; F:DNA binding; C:extracellular region; C:nucleus; C:thylakoid; P:cellular protein modification process; C:cellular_component; P:response to stress; P:cellular process; C:plasma membrane | | IPR005818 (SMART); IPR005818 (PFAM); IPR011991 (G3DSA:1.10.10.GENE3D); SSF46785 (SUPERFAMILY) |
| CA631588 | -85.08 | 8.51E-06 | ---NA--- | | 552 | 0 | - | | no IPS match |
| CK203308 | -85.04 | 8.3E-06 | ---NA--- | | 700 | 0 | - | | no IPS match |
| BE515349 | -75.91 | 6.65E-07 | cytochrome p450 like_tbp | | 335 | 2 | F:catalytic activity; P:metabolic process | | no IPS match |
| TC434513 | -75.67 | 1.35E-06 | bahd acyltransferase dcr-like | | 594 | 8 | F:transferase activity; P:biosynthetic process; P:anatomical structure morphogenesis; P:multicellular organismal development; P:biological_process; P:cellular component organization; P:cell differentiation; C:cytosol | | no IPS match |
| CJ616829 | -75.1 | 2.45E-06 | senescence-associated protein | | 495 | 1 | C:membrane | | no IPS match |
| TC408076 | -72.46 | 1.42E-06 | ---NA--- | | 775 | 0 | - | | no IPS match |
| TC441390 | -67.65 | 2.28E-07 | senescence-associated protein | | 845 | 1 | C:membrane | | IPR002403 (PRINTS); IPR001128 (G3DSA:1.10.630.GENE3D); IPR001128 (PFAM); PTHR24282 (PANTHER); PTHR24282:SF12 (PANTHER); IPR001128 (SUPERFAMILY) |
| TC413011 | -63.15 | 2.28E-07 | ---NA--- | | 828 | 0 | - | | Coil (COILS); IPR001419 (PRINTS); IPR001419 (PFAM); NON_CYTOPLASMIC_DOMAIN (PHOBIUS); SIGNAL_PEPTIDE_H_REGION (PHOBIUS); SIGNAL_PEPTIDE_N_REGION (PHOBIUS); SIGNAL_PEPTIDE (PHOBIUS); SIGNAL_PEPTIDE_C_REGION (PHOBIUS); SignalP-noTM (SIGNALP_GRAM_NEGATIVE); SignalP-TM (SIGNALP_GRAM_POSITIVE); SignalP-noTM (SIGNALP_EUK); IPR016140 (SUPERFAMILY); TMhelix (TMHMM) |
| TC381724 | -62.69 | 1.54E-07 | ferredoxin--nadp leaf isozyme | | 1119 | 4 | F:catalytic activity; F:nucleotide binding; P:metabolic process; C:plastid | | IPR001954 (PRINTS); IPR001376 (PRINTS); IPR016140 (SMART); IPR013771 (G3DSA:1.10.120.GENE3D); IPR013771 (G3DSA:1.10.120.GENE3D); IPR016140 (PFAM); IPR016140 (SUPERFAMILY) |
| TC399665 | -55.89 | 1.42E-06 | gamma-gliadin | | 1222 | 1 | F:molecular_function | | IPR027417 (G3DSA:3.40.50.GENE3D); IPR004125 (G3DSA:1.10.260.GENE3D); IPR004125 (PFAM); IPR000897 (PFAM); PTHR11564 (PANTHER); IPR000897 (PROSITE_PATTERNS); IPR004125 (SUPERFAMILY) |
| CA712118 | -52.4 | 1.67E-06 | ---NA--- | | 290 | 0 | - | | no IPS match |
| CD897082 | -45.49 | 3.29E-06 | ---NA--- | | 288 | 0 | - | | no IPS match |
| TC368707 | -43.21 | 1.9E-06 | low-molecular-weight glutenin partial | | 1452 | 1 | F:molecular_function | | no IPS match |
| TC368810 | -40.6 | 0.000008 | seed maturation protein | | 1196 | 1 | C:membrane | | NON_CYTOPLASMIC_DOMAIN (PHOBIUS); TRANSMEMBRANE (PHOBIUS); CYTOPLASMIC_DOMAIN (PHOBIUS); TMhelix (TMHMM) |
| TC436162 | -38.89 | 1.06E-06 | galactinol--sucrose galactosyltransferase 2 | | 477 | 2 | P:metabolic process; F:catalytic activity | | no IPS match |
| CD913078 | -37.58 | 8.3E-06 | ---NA--- | | 340 | 0 | - | | SignalP-noTM (SIGNALP_EUK) |
| TC432626 | -36.7 | 6.37E-07 | ---NA--- | | 495 | 0 | - | | no IPS match |
| BJ294338 | -36.18 | 3.51E-06 | plasminogen activator inhibitor 1 rna-binding | | 588 | 0 | - | | no IPS match |
| BJ309308 | -34.74 | 7.76E-06 | 60s ribosomal protein l6 | | 698 | 4 | C:ribosome; F:structural molecule activity; P:translation; P:biological_process | | no IPS match |
| TC454289 | -33.81 | 1.35E-07 | ---NA--- | | 228 | 0 | - | | IPR006183 (PRINTS); IPR006115 (PFAM); IPR006114 (PFAM); IPR013328 (G3DSA:1.10.1040.GENE3D); IPR016040 (G3DSA:3.40.50.GENE3D); PTHR11811 (PANTHER); PTHR11811:SF27 (PANTHER); SIGNAL_PEPTIDE_H_REGION (PHOBIUS); SIGNAL_PEPTIDE (PHOBIUS); NON_CYTOPLASMIC_DOMAIN (PHOBIUS); SIGNAL_PEPTIDE_N_REGION (PHOBIUS); SIGNAL_PEPTIDE_C_REGION (PHOBIUS); SSF51735 (SUPERFAMILY); IPR008927 (SUPERFAMILY) |
| CA698189 | -32.07 | 2.62E-06 | ---NA--- | | 490 | 0 | - | | IPR008207 (SUPERFAMILY) |
| TC435585 | -31.59 | 5.41E-07 | lipoxygenase homology domain-containing protein 1-like | | 535 | 1 | C:cytoplasm | | IPR013201 (PFAM); G3DSA:3.90.70.10 (GENE3D); IPR013128 (PANTHER); PTHR12411:SF293 (PANTHER); SSF54001 (SUPERFAMILY) |
| CA668693 | -31.02 | 1.14E-06 | ---NA--- | | 466 | 0 | - | | SIGNAL_PEPTIDE_N_REGION (PHOBIUS); SIGNAL_PEPTIDE (PHOBIUS); NON_CYTOPLASMIC_DOMAIN (PHOBIUS); SIGNAL_PEPTIDE_H_REGION (PHOBIUS); SIGNAL_PEPTIDE_C_REGION (PHOBIUS) |
| TC450096 | -30.82 | 4.85E-06 | ---NA--- | | 276 | 0 | - | | IPR022251 (PFAM) |
| BJ240197 | -30.23 | 2.65E-08 | cytochrome p450 liketbp | | 633 | 0 | - | | IPR013771 (G3DSA:1.10.120.GENE3D); IPR016140 (PFAM); SIGNAL_PEPTIDE_C_REGION (PHOBIUS); SIGNAL_PEPTIDE_H_REGION (PHOBIUS); SIGNAL_PEPTIDE (PHOBIUS); NON_CYTOPLASMIC_DOMAIN (PHOBIUS); SIGNAL_PEPTIDE_N_REGION (PHOBIUS); SignalP-noTM (SIGNALP_GRAM_NEGATIVE); SignalP-noTM (SIGNALP_EUK); SignalP-TM (SIGNALP_GRAM_POSITIVE); IPR016140 (SUPERFAMILY) |
| CA633111 | -28.75 | 1.08E-06 | ---NA--- | | 629 | 0 | - | | no IPS match |
| CA633851 | -28.63 | 1.35E-07 | ---NA--- | | 501 | 0 | - | | no IPS match |
| TC459382 | -28.26 | 5.37E-07 | lipoxygenase homology domain-containing protein 1-like | | 730 | 1 | C:cytoplasm | | G3DSA:3.75.10.10 (GENE3D); IPR007466 (PFAM); PTHR31377 (PANTHER); PTHR31377:SF0 (PANTHER); SSF55909 (SUPERFAMILY) |
| TC447207 | -27.24 | 4.56E-07 | ribulose large partial partial | | 1648 | 10 | P:biosynthetic process; P:carbohydrate metabolic process; P:photosynthesis; F:catalytic activity; F:binding; P:metabolic process; F:nucleic acid binding; F:nucleotide binding; C:plastid; P:cellular process | | IPR002653 (SMART); IPR000058 (SMART); IPR002653 (PFAM); IPR000058 (G3DSA:4.10.1110.GENE3D); IPR000058 (PFAM); PTHR10634:SF16 (PANTHER); PTHR10634 (PANTHER); IPR000058 (PROSITE_PROFILES); IPR002653 (PROSITE_PROFILES); SSF57716 (SUPERFAMILY); SSF118310 (SUPERFAMILY) |
| TC421073 | -27.18 | 1.35E-07 | ---NA--- | | 749 | 0 | - | | IPR001376 (PRINTS); IPR001954 (PRINTS); IPR013771 (G3DSA:1.10.120.GENE3D); IPR016140 (PFAM); IPR016140 (SUPERFAMILY) |
| TC456426 | -27.06 | 2.11E-07 | ---NA--- | | 637 | 0 | - | | Coil (COILS); IPR001954 (PRINTS); IPR016140 (SMART); IPR013771 (G3DSA:1.10.120.GENE3D); IPR016140 (PFAM); IPR013771 (G3DSA:1.10.120.GENE3D); IPR016140 (SUPERFAMILY) |
| CK158331 | -27.04 | 3.64E-06 | chlorophyll a-b binding protein | | 511 | 7 | F:binding; P:generation of precursor metabolites and energy; P:photosynthesis; C:membrane; C:thylakoid; P:cellular protein modification process; C:plastid | | SIGNAL_PEPTIDE (PHOBIUS); NON_CYTOPLASMIC_DOMAIN (PHOBIUS); SIGNAL_PEPTIDE_C_REGION (PHOBIUS); SIGNAL_PEPTIDE_N_REGION (PHOBIUS); SIGNAL_PEPTIDE_H_REGION (PHOBIUS); SignalP-TM (SIGNALP_GRAM_POSITIVE) |
| CA500116 | -26.84 | 1.54E-07 | ---NA--- | | 166 | 0 | - | | IPR012850 (SMART); IPR013781 (G3DSA:3.20.20.GENE3D); IPR012850 (PFAM); IPR013780 (G3DSA:2.60.40.GENE3D); IPR006047 (PFAM); PTHR10357:SF112 (PANTHER); IPR015902 (PANTHER); SSF51011 (SUPERFAMILY); IPR017853 (SUPERFAMILY) |
| CK172288 | -26.8 | 1.06E-06 | ---NA--- | | 921 | 0 | - | | no IPS match |
| TC377364 | -26.76 | 6.37E-07 | s-adenosylmethionine decarboxylase | | 1056 | 5 | P:biosynthetic process; P:nucleobase-containing compound metabolic process; P:cellular process; F:catalytic activity; P:metabolic process | | no IPS match |
| TC438437 | -25.77 | 2.45E-06 | senescence-associated protein | | 896 | 1 | C:membrane | | no IPS match |
| CK153998 | -25.64 | 2.28E-07 | fiber protein fb34 | | 606 | 2 | C:cytoplasm; C:membrane | | no IPS match |
| CD892068 | -25.21 | 9.57E-07 | ---NA--- | | 667 | 0 | - | | no IPS match |
| AL820726 | -25.09 | 6.99E-06 | senescence-associated protein | | 879 | 0 | - | | IPR004911 (PFAM); IPR012336 (G3DSA:3.40.30.GENE3D); PTHR13234:SF8 (PANTHER); IPR004911 (PANTHER); IPR012336 (SUPERFAMILY) |
| TC378473 | -24.6 | 2.83E-07 | exocyst complex component exo84b-like | | 1120 | 2 | F:hydrolase activity; P:metabolic process | | IPR001954 (PRINTS); IPR001376 (PRINTS); IPR013771 (G3DSA:1.10.120.GENE3D); IPR016140 (PFAM); IPR016140 (SUPERFAMILY) |
| CA641136 | -24.51 | 8.3E-06 | ---NA--- | | 263 | 0 | - | | IPR004240 (PFAM); PTHR10766:SF38 (PANTHER); IPR004240 (PANTHER); NON_CYTOPLASMIC_DOMAIN (PHOBIUS); TRANSMEMBRANE (PHOBIUS); CYTOPLASMIC_DOMAIN (PHOBIUS); NON_CYTOPLASMIC_DOMAIN (PHOBIUS); TRANSMEMBRANE (PHOBIUS); CYTOPLASMIC_DOMAIN (PHOBIUS); TRANSMEMBRANE (PHOBIUS); CYTOPLASMIC_DOMAIN (PHOBIUS); TRANSMEMBRANE (PHOBIUS); TMhelix (TMHMM); TMhelix (TMHMM); TMhelix (TMHMM); TMhelix (TMHMM) |
| TC411260 | -24.31 | 6.87E-06 | protein breast cancer susceptibility 1-like protein | | 678 | 6 | P:DNA repair; F:zinc ion binding; P:protein ubiquitination; F:ubiquitin-protein transferase activity; C:nucleus; F:DNA binding | | no IPS match |
| TC422094 | -23.78 | 1.07E-06 | eukaryotic initiation factor 4a-1 | | 1042 | 5 | F:hydrolase activity; F:nucleotide binding; F:translation factor activity, nucleic acid binding; C:ribosome; P:translation | | NON_CYTOPLASMIC_DOMAIN (PHOBIUS); SIGNAL_PEPTIDE_C_REGION (PHOBIUS); SIGNAL_PEPTIDE_H_REGION (PHOBIUS); SIGNAL_PEPTIDE_N_REGION (PHOBIUS); SIGNAL_PEPTIDE (PHOBIUS); SignalP-TM (SIGNALP_GRAM_POSITIVE) |
| CA639229 | -23.43 | 7.28E-06 | ---NA--- | | 468 | 0 | - | | IPR000504 (SMART); PF14259 (PFAM); IPR012677 (G3DSA:3.30.70.GENE3D); PTHR12999 (PANTHER); PTHR12999:SF15 (PANTHER); IPR000504 (PROSITE_PROFILES); SSF54928 (SUPERFAMILY) |
| TC434913 | -22.57 | 2.1E-06 | ---NA--- | | 404 | 0 | - | | no IPS match |
| CA610082 | -22.04 | 1.14E-06 | ---NA--- | | 434 | 0 | - | | SIGNAL_PEPTIDE (PHOBIUS); SIGNAL_PEPTIDE_N_REGION (PHOBIUS); SIGNAL_PEPTIDE_H_REGION (PHOBIUS); NON_CYTOPLASMIC_DOMAIN (PHOBIUS); SIGNAL_PEPTIDE_C_REGION (PHOBIUS); SignalP-noTM (SIGNALP_EUK); SignalP-TM (SIGNALP_GRAM_POSITIVE); SignalP-noTM (SIGNALP_GRAM_NEGATIVE) |
| TC423161 | -21.5 | 3.38E-07 | chlorophyll a-b binding protein cp24 chloroplastic | | 700 | 4 | C:membrane; P:photosynthesis; C:thylakoid; C:plastid | | IPR029044 (G3DSA:3.90.550.GENE3D); IPR002495 (PFAM); IPR029993 (PANTHER); PTHR32116:SF12 (PANTHER); IPR029044 (SUPERFAMILY) |
| TC451876 | -21.13 | 2.49E-06 | ---NA--- | | 513 | 0 | - | | SIGNAL_PEPTIDE_C_REGION (PHOBIUS); SIGNAL_PEPTIDE (PHOBIUS); SIGNAL_PEPTIDE_H_REGION (PHOBIUS); NON_CYTOPLASMIC_DOMAIN (PHOBIUS); SIGNAL_PEPTIDE_N_REGION (PHOBIUS); SignalP-noTM (SIGNALP_EUK); SignalP-noTM (SIGNALP_GRAM_NEGATIVE); SignalP-TM (SIGNALP_GRAM_POSITIVE) |
| TC375013 | -21.08 | 7.94E-06 | nadh-plastoquinone oxidoreductase subunit k | | 1721 | 12 | C:plasma membrane; F:binding; F:catalytic activity; P:generation of precursor metabolites and energy; P:photosynthesis; C:membrane; C:plastid; C:thylakoid; P:nucleobase-containing compound metabolic process; P:biosynthetic process; P:cellular process; P:transport | | TRANSMEMBRANE (PHOBIUS); CYTOPLASMIC_DOMAIN (PHOBIUS); NON_CYTOPLASMIC_DOMAIN (PHOBIUS) |
| TC404906 | -20.9 | 9.73E-06 | chaperone protein dnaj 10-like | | 649 | 0 | - | | Coil (COILS); Coil (COILS); IPR006016 (PFAM); IPR014729 (G3DSA:3.40.50.GENE3D); PTHR31964 (PANTHER); SSF52402 (SUPERFAMILY) |
| TC420361 | -20.72 | 5.03E-07 | hypothetical protein TRIUR3_13515 | | 506 | 2 | C:cytoplasm; C:mitochondrion | | IPR029149 (G3DSA:3.40.350.GENE3D); PTHR10804:SF87 (PANTHER); PTHR10804:SF87 (PANTHER); PTHR10804 (PANTHER) |
| TC437369 | -20.71 | 2.63E-07 | nadh dehydrogenase | | 622 | 14 | P:carbohydrate metabolic process; P:catabolic process; P:protein metabolic process; P:cellular process; F:catalytic activity; C:membrane; C:mitochondrion; P:response to stress; C:plastid; P:cellular component organization; P:biosynthetic process; P:generation of precursor metabolites and energy; P:nucleobase-containing compound metabolic process; P:transport | | IPR011991 (G3DSA:1.10.10.GENE3D); IPR024550 (PFAM); PTHR12949 (PANTHER) |
| TC448847 | -20.58 | 6.98E-07 | cysteine proteinase 1-like | | 1122 | 3 | C:cytoplasm; P:protein metabolic process; F:hydrolase activity | | no IPS match |
| TC405912 | -20.3 | 1.35E-07 | harpin-induced family | | 754 | 1 | C:mitochondrion | | no IPS match |
| TC387353 | -20.01 | 1.07E-06 | histone h4 | | 700 | 17 | C:cytosol; C:intracellular; P:response to stress; P:response to abiotic stimulus; C:mitochondrion; C:nucleolus; C:cellular_component; F:DNA binding; C:membrane; C:vacuole; C:thylakoid; P:cellular component organization; P:DNA metabolic process; F:protein binding; C:plastid; C:Golgi apparatus; C:plasma membrane | | no IPS match |
| TC371597 | -19.93 | 8.3E-06 | low temperature-responsive rna-binding protein | | 1020 | 8 | P:response to stress; P:response to abiotic stimulus; P:biosynthetic process; P:nucleobase-containing compound metabolic process; F:RNA binding; F:DNA binding; P:response to endogenous stimulus; F:nucleotide binding | | no IPS match |
| GH731673 | -19.79 | 1.14E-06 | zinc finger protein 2 | | 700 | 1 | F:metal ion binding | | IPR014718 (G3DSA:2.70.98.GENE3D); IPR008183 (PFAM); PTHR10091 (PANTHER); PTHR10091:SF3 (PANTHER); IPR011013 (SUPERFAMILY) |
| TC414628 | -19.76 | 4.49E-06 | udp-glucuronic acid decarboxylase 6-like isoform x1 | | 687 | 4 | C:cytoplasm; P:metabolic process; F:catalytic activity; F:binding | | no IPS match |
| TC421494 | -19.63 | 3.83E-06 | ---NA--- | | 714 | 0 | - | | no IPS match |
| CA498547 | -19.42 | 1.35E-07 | ---NA--- | | 249 | 0 | - | | SIGNAL_PEPTIDE_H_REGION (PHOBIUS); NON_CYTOPLASMIC_DOMAIN (PHOBIUS); SIGNAL_PEPTIDE_C_REGION (PHOBIUS); SIGNAL_PEPTIDE (PHOBIUS); SIGNAL_PEPTIDE_N_REGION (PHOBIUS); TMhelix (TMHMM) |
| CD922604 | -19.17 | 2.43E-06 | ---NA--- | | 308 | 0 | - | | PF13410 (PFAM); IPR012336 (G3DSA:3.40.30.GENE3D); IPR010987 (G3DSA:1.20.1050.GENE3D); IPR004045 (PFAM); PTHR11260:SF174 (PANTHER); PTHR11260 (PANTHER); IPR010987 (PROSITE_PROFILES); IPR004045 (PROSITE_PROFILES); IPR010987 (SUPERFAMILY); IPR012336 (SUPERFAMILY) |
| CA648080 | -18.25 | 1.41E-06 | ---NA--- | | 239 | 0 | - | | IPR005349 (PFAM); IPR005349 (PANTHER); PTHR12668:SF12 (PANTHER); TRANSMEMBRANE (PHOBIUS); TRANSMEMBRANE (PHOBIUS); CYTOPLASMIC_DOMAIN (PHOBIUS); TRANSMEMBRANE (PHOBIUS); NON_CYTOPLASMIC_DOMAIN (PHOBIUS); NON_CYTOPLASMIC_DOMAIN (PHOBIUS); CYTOPLASMIC_DOMAIN (PHOBIUS); CYTOPLASMIC_DOMAIN (PHOBIUS); TRANSMEMBRANE (PHOBIUS); TMhelix (TMHMM); TMhelix (TMHMM); TMhelix (TMHMM); TMhelix (TMHMM) |
| TC417675 | -18.1 | 2.42E-06 | histone -like isoform 2 | | 668 | 4 | F:protein binding; C:intracellular; C:nucleus; F:DNA binding | | no IPS match |
| CA697848 | -17.83 | 7.42E-07 | ---NA--- | | 586 | 0 | - | | no IPS match |
| TC439372 | -17.13 | 2.21E-07 | ---NA--- | | 497 | 0 | - | | no IPS match |
| TC440231 | -17.11 | 1.42E-06 | ---NA--- | | 588 | 0 | - | | no IPS match |
| TC416103 | -16.87 | 3.29E-06 | signal peptidase complex subunit 1 | | 576 | 7 | F:hydrolase activity; C:cytoplasm; P:protein metabolic process; P:cellular process; C:membrane; C:mitochondrion; C:endoplasmic reticulum | | IPR024553 (PFAM); PTHR31373 (PANTHER); IPR002035 (SUPERFAMILY) |
| TC380174 | -16.77 | 6.44E-07 | senescence-associated protein | | 1108 | 1 | C:membrane | | IPR000814 (PRINTS); IPR012295 (G3DSA:3.30.310.GENE3D); IPR000814 (PFAM); IPR000814 (PANTHER); IPR030491 (PROSITE_PATTERNS); IPR030491 (PROSITE_PATTERNS); IPR000814 (HAMAP); SSF55945 (SUPERFAMILY); SSF55945 (SUPERFAMILY) |
| TC426484 | -16.73 | 9.73E-06 | ---NA--- | | 461 | 0 | - | | no IPS match |
| TC443845 | -16.52 | 8.51E-06 | ---NA--- | | 348 | 0 | - | | IPR016482 (PFAM); IPR030671 (PANTHER); CYTOPLASMIC_DOMAIN (PHOBIUS); TRANSMEMBRANE (PHOBIUS); NON_CYTOPLASMIC_DOMAIN (PHOBIUS); TMhelix (TMHMM) |
| TC385169 | -16.48 | 8.84E-06 | histone h2a | | 730 | 4 | F:protein binding; C:intracellular; C:nucleus; F:DNA binding | | no IPS match |
| TC390498 | -16.4 | 3.54E-06 | histone h4 | | 688 | 17 | C:cytosol; C:intracellular; P:response to stress; P:response to abiotic stimulus; C:mitochondrion; C:nucleolus; C:cellular_component; F:DNA binding; C:membrane; C:vacuole; C:thylakoid; P:cellular component organization; P:DNA metabolic process; F:protein binding; C:plastid; C:Golgi apparatus; C:plasma membrane | | no IPS match |
| TC374373 | -16.31 | 1.17E-06 | hypothetical protein F775_07910 | | 1396 | 5 | F:metal ion binding; C:vacuole; F:hydrolase activity; P:metabolic process; C:cytosol | | IPR000626 (SMART); IPR022617 (PFAM); G3DSA:3.10.20.90 (GENE3D); PTHR10562 (PANTHER); IPR000626 (PROSITE_PROFILES); IPR029071 (SUPERFAMILY) |
| TC451770 | -16.04 | 8.63E-08 | ---NA--- | | 255 | 0 | - | | IPR001954 (PRINTS); IPR001376 (PRINTS); IPR016140 (SMART); IPR013771 (G3DSA:1.10.120.GENE3D); IPR016140 (PFAM); IPR013771 (G3DSA:1.10.120.GENE3D); SIGNAL_PEPTIDE_C_REGION (PHOBIUS); SIGNAL_PEPTIDE_H_REGION (PHOBIUS); SIGNAL_PEPTIDE (PHOBIUS); NON_CYTOPLASMIC_DOMAIN (PHOBIUS); SIGNAL_PEPTIDE_N_REGION (PHOBIUS); SignalP-noTM (SIGNALP_GRAM_NEGATIVE); SignalP-noTM (SIGNALP_EUK); SignalP-TM (SIGNALP_GRAM_POSITIVE); IPR016140 (SUPERFAMILY) |
| TC454476 | -15.85 | 5.09E-07 | ---NA--- | | 185 | 0 | - | | NON_CYTOPLASMIC_DOMAIN (PHOBIUS); TRANSMEMBRANE (PHOBIUS); CYTOPLASMIC_DOMAIN (PHOBIUS); TMhelix (TMHMM) |
| TC444968 | -15.57 | 5.63E-06 | ---NA--- | | 321 | 0 | - | | no IPS match |
| TC433645 | -15.41 | 2.75E-06 | ---NA--- | | 540 | 0 | - | | G3DSA:1.10.287.70 (GENE3D); IPR013833 (G3DSA:1.20.120.GENE3D); IPR000298 (PFAM); PTHR11403:SF3 (PANTHER); IPR024791 (PANTHER); TRANSMEMBRANE (PHOBIUS); CYTOPLASMIC_DOMAIN (PHOBIUS); TRANSMEMBRANE (PHOBIUS); NON_CYTOPLASMIC_DOMAIN (PHOBIUS); TRANSMEMBRANE (PHOBIUS); TRANSMEMBRANE (PHOBIUS); NON_CYTOPLASMIC_DOMAIN (PHOBIUS); NON_CYTOPLASMIC_DOMAIN (PHOBIUS); CYTOPLASMIC_DOMAIN (PHOBIUS); TRANSMEMBRANE (PHOBIUS); CYTOPLASMIC_DOMAIN (PHOBIUS); NON_CYTOPLASMIC_DOMAIN (PHOBIUS); TRANSMEMBRANE (PHOBIUS); IPR000298 (PROSITE_PROFILES); IPR000298 (SUPERFAMILY); TMhelix (TMHMM); TMhelix (TMHMM); TMhelix (TMHMM); TMhelix (TMHMM); TMhelix (TMHMM); TMhelix (TMHMM) |
| TC425178 | -14.99 | 2.22E-06 | amino acid permease 8-like | | 619 | 1 | C:membrane | | no IPS match |
| CK197053 | -14.88 | 1.35E-07 | ---NA--- | | 732 | 0 | - | | no IPS match |
| TC439160 | -14.74 | 1.14E-06 | protein | | 1408 | 4 | P:response to abiotic stimulus; P:response to stress; C:membrane; C:plastid | | SignalP-noTM (SIGNALP_EUK) |
| TC454154 | -14.72 | 1.32E-06 | disease resistance response protein 206-like | | 931 | 1 | C:cytoplasm | | IPR001147 (PFAM); IPR001147 (G3DSA:2.30.30.GENE3D); IPR001147 (PANTHER); IPR018259 (PROSITE_PATTERNS); IPR008991 (SUPERFAMILY) |
| BJ319889 | -14.53 | 1.35E-06 | senescence-associated protein | | 706 | 0 | - | | no IPS match |
| TC455528 | -14.36 | 5.39E-06 | ---NA--- | | 228 | 0 | - | | CYTOPLASMIC_DOMAIN (PHOBIUS); TRANSMEMBRANE (PHOBIUS); NON_CYTOPLASMIC_DOMAIN (PHOBIUS); TMhelix (TMHMM) |
| TC401022 | -14.32 | 4.57E-06 | loc100283229 precursor | | 755 | 7 | F:binding; C:cytoplasm; F:molecular_function; P:biosynthetic process; P:carbohydrate metabolic process; P:cellular process; C:membrane | | G3DSA:2.40.30.10 (GENE3D); IPR004160 (PFAM); G3DSA:2.40.30.10 (GENE3D); PTHR23115:SF124 (PANTHER); PTHR23115 (PANTHER); IPR009000 (SUPERFAMILY); IPR009001 (SUPERFAMILY) |
| TC429650 | -13.97 | 2.8E-06 | ---NA--- | | 407 | 0 | - | | IPR002401 (PRINTS); IPR001128 (PRINTS); IPR001128 (PFAM); IPR001128 (G3DSA:1.10.630.GENE3D); PTHR24298:SF58 (PANTHER); PTHR24298 (PANTHER); IPR017972 (PROSITE_PATTERNS); IPR001128 (SUPERFAMILY) |
| CA737330 | -13.92 | 1.14E-06 | ---NA--- | | 578 | 0 | - | | IPR000782 (SMART); IPR000782 (G3DSA:2.30.180.GENE3D); IPR000782 (PFAM); PTHR32382:SF4 (PANTHER); PTHR32382 (PANTHER); CYTOPLASMIC_DOMAIN (PHOBIUS); SIGNAL_PEPTIDE_H_REGION (PHOBIUS); SIGNAL_PEPTIDE_C_REGION (PHOBIUS); SIGNAL_PEPTIDE (PHOBIUS); SIGNAL_PEPTIDE_N_REGION (PHOBIUS); NON_CYTOPLASMIC_DOMAIN (PHOBIUS); TRANSMEMBRANE (PHOBIUS); IPR000782 (PROSITE_PROFILES); SignalP-noTM (SIGNALP_EUK); SignalP-noTM (SIGNALP_GRAM_NEGATIVE); SignalP-TM (SIGNALP_GRAM_POSITIVE); IPR000782 (SUPERFAMILY); TMhelix (TMHMM); TMhelix (TMHMM) |
| CK168985 | -13.9 | 0.000002 | 60s ribosomal protein l21 | | 543 | 5 | C:ribosome; F:structural molecule activity; P:translation; C:mitochondrion; P:biological_process | | Coil (COILS); IPR009071 (SMART); IPR009071 (PFAM); IPR009071 (G3DSA:1.10.30.GENE3D); PTHR13711:SF167 (PANTHER); PTHR13711 (PANTHER); IPR009071 (PROSITE_PROFILES); IPR009071 (SUPERFAMILY) |
| CK167046 | -13.9 | 8.15E-06 | elongation factor 1-alpha | | 1165 | 6 | F:translation factor activity, nucleic acid binding; F:nucleotide binding; F:transferase activity; F:hydrolase activity; C:ribosome; P:translation | | no IPS match |
| TC382900 | -13.64 | 3.27E-06 | histone h2a family protein | | 863 | 4 | F:protein binding; C:intracellular; C:nucleus; F:DNA binding | | no IPS match |
| TC414718 | -13.63 | 5.78E-06 | beta-expansin 1a precursor | | 699 | 7 | C:extracellular region; C:membrane; C:cytoplasm; P:cellular component organization; P:cellular process; P:reproduction; C:cell wall | | IPR008733 (PFAM); PTHR12652:SF17 (PANTHER); PTHR12652 (PANTHER) |
| AL818129 | -13.58 | 5.78E-06 | ---NA--- | | 463 | 0 | - | | no IPS match |
| TC456249 | -13.5 | 6.25E-06 | cysteine proteinase inhibitor 10-like | | 675 | 4 | P:protein metabolic process; P:biological_process; C:cytoplasm; F:enzyme regulator activity | | SIGNAL_PEPTIDE_N_REGION (PHOBIUS); SIGNAL_PEPTIDE_C_REGION (PHOBIUS); SIGNAL_PEPTIDE (PHOBIUS); NON_CYTOPLASMIC_DOMAIN (PHOBIUS); SIGNAL_PEPTIDE_H_REGION (PHOBIUS); SignalP-TM (SIGNALP_GRAM_POSITIVE); SignalP-noTM (SIGNALP_EUK) |
| TC438213 | -13.35 | 2.35E-06 | ---NA--- | | 429 | 0 | - | | no IPS match |
| TC416021 | -13.04 | 2.31E-06 | subtilisin-like protease sdd1-like | | 1390 | 4 | C:cytoplasm; C:cell wall; P:protein metabolic process; F:hydrolase activity | | NON_CYTOPLASMIC_DOMAIN (PHOBIUS); TRANSMEMBRANE (PHOBIUS); CYTOPLASMIC_DOMAIN (PHOBIUS); CYTOPLASMIC_DOMAIN (PHOBIUS); TRANSMEMBRANE (PHOBIUS); TMhelix (TMHMM) |
| TC425686 | -12.89 | 3.6E-06 | lariat debranching enzyme | | 584 | 4 | P:embryo development; P:nucleobase-containing compound metabolic process; F:nuclease activity; C:nucleus | | IPR002048 (SMART); IPR011992 (G3DSA:1.10.238.GENE3D); IPR011992 (G3DSA:1.10.238.GENE3D); IPR011992 (PFAM); PTHR23050:SF136 (PANTHER); PTHR23050 (PANTHER); IPR018247 (PROSITE_PATTERNS); IPR018247 (PROSITE_PATTERNS); IPR018247 (PROSITE_PATTERNS); IPR018247 (PROSITE_PATTERNS); IPR002048 (PROSITE_PROFILES); IPR002048 (PROSITE_PROFILES); IPR002048 (PROSITE_PROFILES); IPR002048 (PROSITE_PROFILES); SSF47473 (SUPERFAMILY) |
| TC411761 | -12.87 | 9.31E-06 | ---NA--- | | 561 | 0 | - | | Coil (COILS); Coil (COILS); IPR004882 (PFAM); IPR004882 (PANTHER); PTHR12375:SF18 (PANTHER) |
| AL830834 | -12.84 | 3.47E-06 | histone h2ax-like | | 479 | 8 | P:nucleobase-containing compound metabolic process; C:intracellular; C:nucleolus; F:DNA binding; P:cellular component organization; P:DNA metabolic process; F:protein binding; P:biological_process | | IPR023626 (G3DSA:1.10.1620.GENE3D); IPR000077 (PFAM); IPR000077 (PANTHER); IPR020083 (PROSITE_PATTERNS); NON_CYTOPLASMIC_DOMAIN (PHOBIUS); SIGNAL_PEPTIDE (PHOBIUS); SIGNAL_PEPTIDE_C_REGION (PHOBIUS); SIGNAL_PEPTIDE_N_REGION (PHOBIUS); SIGNAL_PEPTIDE_H_REGION (PHOBIUS); IPR023626 (SUPERFAMILY) |
| CA679310 | -12.8 | 1.14E-06 | ---NA--- | | 474 | 0 | - | | SignalP-TM (SIGNALP_GRAM_POSITIVE) |
| BE515440 | -12.77 | 3.48E-06 | ---NA--- | | 439 | 0 | - | | no IPS match |
| CA635637 | -12.73 | 7.74E-06 | ---NA--- | | 349 | 0 | - | | no IPS match |
| TC427527 | -12.58 | 6.98E-07 | protein mother of ft and tf 1-like | | 843 | 0 | - | | no IPS match |
| TC399832 | -12.48 | 5.63E-07 | beta-expansin 1a precursor | | 867 | 7 | C:extracellular region; C:membrane; C:cytoplasm; P:cellular component organization; P:cellular process; P:reproduction; C:cell wall | | IPR015916 (G3DSA:2.130.10.GENE3D); PTHR24412:SF0 (PANTHER); PTHR24412 (PANTHER); SSF117281 (SUPERFAMILY) |
| TC440606 | -12.47 | 1.14E-06 | ---NA--- | | 468 | 0 | - | | no IPS match |
| TC404223 | -12.45 | 1.88E-06 | hypothetical protein TRIUR3_11328 | | 602 | 0 | - | | no IPS match |
| CK196988 | -12.42 | 1.56E-06 | ---NA--- | | 677 | 0 | - | | IPR020471 (PRINTS); IPR023210 (G3DSA:3.20.20.GENE3D); IPR023210 (PFAM); PTHR11732:SF142 (PANTHER); IPR001395 (PANTHER); IPR018170 (PROSITE_PATTERNS); IPR023210 (SUPERFAMILY) |
| TC378628 | -12.27 | 2.28E-07 | 40s ribosomal protein s10-like | | 985 | 1 | C:ribosome | | IPR000864 (PRINTS); G3DSA:3.30.10.10 (GENE3D); IPR000864 (PFAM); IPR000864 (PROSITE_PATTERNS); IPR000864 (SUPERFAMILY) |
| TC400373 | -12.17 | 2.45E-06 | embr-h2 protein expressed | | 653 | 5 | C:cell; C:membrane; C:cytoplasm; F:binding; C:intracellular | | no IPS match |
| TC415025 | -12.11 | 8.63E-08 | histone h4 | | 1281 | 17 | C:cytosol; C:intracellular; P:response to stress; P:response to abiotic stimulus; C:mitochondrion; C:nucleolus; C:cellular_component; F:DNA binding; C:membrane; C:vacuole; C:thylakoid; P:cellular component organization; P:DNA metabolic process; F:protein binding; C:plastid; C:Golgi apparatus; C:plasma membrane | | TRANSMEMBRANE (PHOBIUS); NON_CYTOPLASMIC_DOMAIN (PHOBIUS); CYTOPLASMIC_DOMAIN (PHOBIUS) |
| TC380977 | -11.87 | 3.08E-06 | hypothetical protein F775_31117 | | 766 | 0 | - | | no IPS match |
| TC447091 | -11.84 | 8.81E-06 | ---NA--- | | 340 | 0 | - | | TRANSMEMBRANE (PHOBIUS); NON_CYTOPLASMIC_DOMAIN (PHOBIUS); CYTOPLASMIC_DOMAIN (PHOBIUS); SignalP-TM (SIGNALP_GRAM_POSITIVE) |
| CK211305 | -11.76 | 7.53E-06 | ralfl33 precursor | | 758 | 3 | C:cytoplasm; P:nucleobase-containing compound metabolic process; F:hydrolase activity | | IPR000644 (SMART); IPR000644 (PFAM); IPR013785 (G3DSA:3.20.20.GENE3D); PTHR11911 (PANTHER); PTHR11911:SF51 (PANTHER); IPR000644 (PROSITE_PROFILES); SSF54631 (SUPERFAMILY) |
| CA598201 | -11.75 | 8.15E-06 | ---NA--- | | 618 | 0 | - | | TRANSMEMBRANE (PHOBIUS); CYTOPLASMIC_DOMAIN (PHOBIUS); NON_CYTOPLASMIC_DOMAIN (PHOBIUS); NON_CYTOPLASMIC_DOMAIN (PHOBIUS); CYTOPLASMIC_DOMAIN (PHOBIUS); TRANSMEMBRANE (PHOBIUS); TRANSMEMBRANE (PHOBIUS); TMhelix (TMHMM); TMhelix (TMHMM); TMhelix (TMHMM) |
| TC418183 | -11.51 | 1.32E-06 | chlorophyll a-b binding chloroplastic | | 578 | 7 | F:binding; P:generation of precursor metabolites and energy; P:photosynthesis; C:membrane; C:thylakoid; P:cellular protein modification process; C:plastid | | CYTOPLASMIC_DOMAIN (PHOBIUS); NON_CYTOPLASMIC_DOMAIN (PHOBIUS); TRANSMEMBRANE (PHOBIUS) |
| TC423791 | -11.47 | 5.41E-07 | ---NA--- | | 1240 | 0 | - | | IPR003903 (SMART); IPR003903 (PFAM); IPR027040 (PANTHER); IPR003903 (PROSITE_PROFILES) |
| TC389131 | -11.34 | 1.14E-06 | 40s ribosomal protein s10-like | | 938 | 1 | C:ribosome | | no IPS match |
| TC385207 | -11.28 | 5.62E-06 | histone h2a family protein | | 1063 | 4 | F:protein binding; C:intracellular; C:nucleus; F:DNA binding | | IPR011992 (G3DSA:1.10.238.GENE3D); IPR011992 (PFAM); PTHR12085 (PANTHER); IPR018247 (PROSITE_PATTERNS); IPR002048 (PROSITE_PROFILES); IPR002048 (PROSITE_PROFILES); IPR002048 (PROSITE_PROFILES); SSF47473 (SUPERFAMILY) |
| TC398787 | -11.07 | 2.64E-07 | hypothetical protein F775_05432 | | 812 | 0 | - | | no IPS match |
| CK197528 | -10.93 | 3.06E-06 | ---NA--- | | 255 | 0 | - | | IPR012946 (SMART); PTHR32227:SF56 (PANTHER); PTHR32227 (PANTHER) |
| CA665155 | -10.84 | 1.29E-06 | 40s ribosomal protein s16-like | | 616 | 4 | C:ribosome; F:structural molecule activity; P:translation; P:biological_process | | IPR015880 (SMART); IPR007087 (PFAM); PF13912 (PFAM); IPR013087 (G3DSA:3.30.160.GENE3D); PTHR26374:SF162 (PANTHER); PTHR26374 (PANTHER); IPR007087 (PROSITE_PATTERNS); IPR007087 (PROSITE_PATTERNS); IPR007087 (PROSITE_PROFILES); IPR007087 (PROSITE_PROFILES); SSF57667 (SUPERFAMILY); SSF57667 (SUPERFAMILY) |
| TC441811 | -10.79 | 1.24E-06 | ---NA--- | | 668 | 0 | - | | SignalP-TM (SIGNALP_GRAM_POSITIVE) |
| BJ234824 | -10.65 | 1.74E-06 | ---NA--- | | 319 | 0 | - | | TRANSMEMBRANE (PHOBIUS); CYTOPLASMIC_DOMAIN (PHOBIUS); NON_CYTOPLASMIC_DOMAIN (PHOBIUS); TMhelix (TMHMM) |
| TC452538 | -10.63 | 3.77E-06 | ---NA--- | | 380 | 0 | - | | no IPS match |
| TC406029 | -10.44 | 4.7E-06 | beta- partial | | 792 | 7 | C:cytoplasm; F:structural molecule activity; F:nucleotide binding; P:cellular process; P:cellular component organization; F:hydrolase activity; C:cytoskeleton | | IPR000782 (SMART); IPR000782 (G3DSA:2.30.180.GENE3D); IPR000782 (PFAM); PTHR32382 (PANTHER); PTHR32382:SF4 (PANTHER); NON_CYTOPLASMIC_DOMAIN (PHOBIUS); SIGNAL_PEPTIDE_C_REGION (PHOBIUS); SIGNAL_PEPTIDE_N_REGION (PHOBIUS); SIGNAL_PEPTIDE_H_REGION (PHOBIUS); SIGNAL_PEPTIDE (PHOBIUS); IPR000782 (PROSITE_PROFILES); IPR000782 (PROSITE_PROFILES); SignalP-noTM (SIGNALP_GRAM_NEGATIVE); SignalP-TM (SIGNALP_EUK); IPR000782 (SUPERFAMILY); IPR000782 (SUPERFAMILY); TMhelix (TMHMM) |
| TC368737 | -10.2 | 7.94E-06 | ethylene-responsive transcription factor rap2-4-like | | 1789 | 6 | P:biosynthetic process; P:nucleobase-containing compound metabolic process; F:sequence-specific DNA binding transcription factor activity; F:DNA binding; C:nucleus; C:intracellular | | IPR000232 (PRINTS); IPR000232 (SMART); IPR011991 (G3DSA:1.10.10.GENE3D); IPR000232 (PFAM); IPR027725 (PANTHER); IPR027709 (PTHR10015:PANTHER); IPR000232 (PROSITE_PATTERNS); SSF46785 (SUPERFAMILY) |
| TC446247 | -9.79 | 7.2E-07 | ---NA--- | | 300 | 0 | - | | G3DSA:2.60.260.20 (GENE3D); IPR002939 (PFAM); PTHR24076 (PANTHER); PTHR24076:SF1 (PANTHER); IPR008971 (SUPERFAMILY) |
| TC433323 | -9.65 | 9.31E-06 | hypothetical protein F775_23752 | | 416 | 2 | F:hydrolase activity; P:protein metabolic process | | IPR000823 (PRINTS); IPR002016 (PRINTS); G3DSA:1.10.520.10 (GENE3D); G3DSA:1.10.420.10 (GENE3D); IPR002016 (PFAM); PTHR31235:SF11 (PANTHER); PTHR31235 (PANTHER); IPR019794 (PROSITE_PATTERNS); SIGNAL_PEPTIDE (PHOBIUS); SIGNAL_PEPTIDE_C_REGION (PHOBIUS); NON_CYTOPLASMIC_DOMAIN (PHOBIUS); SIGNAL_PEPTIDE_N_REGION (PHOBIUS); SIGNAL_PEPTIDE_H_REGION (PHOBIUS); IPR002016 (PROSITE_PROFILES); SignalP-TM (SIGNALP_GRAM_POSITIVE); SignalP-noTM (SIGNALP_EUK); SignalP-noTM (SIGNALP_GRAM_NEGATIVE); IPR010255 (SUPERFAMILY) |
| TC450757 | -9.64 | 6.72E-06 | hypothetical protein F775_31117 | | 1028 | 0 | - | | TMhelix (TMHMM) |
| TC417298 | -9.62 | 1.49E-06 | 60s ribosomal protein l12 | | 854 | 4 | C:ribosome; F:structural molecule activity; P:translation; P:biological_process | | no IPS match |
| TC410983 | -9.4 | 9.31E-06 | elongation factor 1-alpha | | 1023 | 9 | F:translation factor activity, nucleic acid binding; F:nucleotide binding; F:transferase activity; F:hydrolase activity; C:ribosome; P:translation; P:nucleobase-containing compound metabolic process; P:metabolic process; P:cellular process | | no IPS match |
| TC409241 | -9.35 | 7.74E-06 | s-adenosylmethionine decarboxylase | | 969 | 5 | P:biosynthetic process; P:nucleobase-containing compound metabolic process; P:cellular process; F:catalytic activity; P:metabolic process | | no IPS match |
| TC446772 | -9.24 | 4.45E-07 | ---NA--- | | 281 | 0 | - | | IPR029004 (PFAM); IPR002672 (PANTHER); PTHR10544:SF0 (PANTHER) |
| TC420062 | -9.07 | 8.51E-06 | 60s ribosomal protein l39 | | 495 | 5 | C:ribosome; F:structural molecule activity; P:translation; C:mitochondrion; P:biological_process | | Coil (COILS); IPR009053 (G3DSA:1.10.287.GENE3D); IPR011599 (TIGRFAM); IPR004127 (PFAM); PTHR12674 (PANTHER); IPR009053 (SUPERFAMILY) |
| TC441874 | -8.94 | 8.84E-06 | pollen-specific leucine-rich repeat extensin-like protein 1-like | | 1077 | 2 | P:transport; F:binding | | Coil (COILS); IPR002452 (PRINTS); IPR018316 (SMART); IPR003008 (G3DSA:3.40.50.GENE3D); IPR023123 (G3DSA:1.10.287.GENE3D); IPR018316 (G3DSA:3.30.1330.GENE3D); IPR018316 (PFAM); PTHR11588:SF48 (PANTHER); IPR000217 (PANTHER); IPR003008 (SUPERFAMILY); IPR008280 (SUPERFAMILY) |
| TC392043 | -8.93 | 4.72E-06 | amino acid permease 3-like | | 918 | 1 | C:membrane | | TRANSMEMBRANE (PHOBIUS); NON_CYTOPLASMIC_DOMAIN (PHOBIUS); TRANSMEMBRANE (PHOBIUS); CYTOPLASMIC_DOMAIN (PHOBIUS); CYTOPLASMIC_DOMAIN (PHOBIUS); SignalP-TM (SIGNALP_GRAM_POSITIVE); TMhelix (TMHMM); TMhelix (TMHMM) |
| TC449858 | -8.93 | 7.74E-06 | s-adenosyl methionine decarboxylase 2 | | 1016 | 4 | P:biosynthetic process; P:cellular process; P:metabolic process; F:catalytic activity | | IPR006501 (PFAM); IPR006501 (G3DSA:1.20.140.GENE3D); NON_CYTOPLASMIC_DOMAIN (PHOBIUS); SIGNAL_PEPTIDE_N_REGION (PHOBIUS); SIGNAL_PEPTIDE_C_REGION (PHOBIUS); SIGNAL_PEPTIDE (PHOBIUS); SIGNAL_PEPTIDE_H_REGION (PHOBIUS); SignalP-noTM (SIGNALP_EUK); IPR006501 (SUPERFAMILY) |
| BG313110 | -8.87 | 5.4E-06 | glycosyltransferase-like domain-containing protein 2-like | | 515 | 2 | F:transferase activity; P:metabolic process | | no IPS match |
| TC445402 | -8.85 | 4.63E-07 | gamma-glutamyltranspeptidase 1 | | 902 | 7 | C:vacuole; F:transferase activity; P:metabolic process; P:cellular process; F:hydrolase activity; C:membrane; P:lipid metabolic process | | no IPS match |
| TC427874 | -8.85 | 8.42E-06 | nac transcription factor | | 573 | 3 | P:biosynthetic process; P:nucleobase-containing compound metabolic process; F:DNA binding | | no IPS match |
| TC412273 | -8.84 | 1.69E-06 | subtilisin-like protease sdd1-like | | 1614 | 4 | C:cytoplasm; C:cell wall; P:protein metabolic process; F:hydrolase activity | | IPR013154 (PFAM); IPR011032 (G3DSA:3.90.180.GENE3D); IPR002085 (PANTHER); PTHR11695:SF451 (PANTHER); IPR002328 (PROSITE_PATTERNS); IPR011032 (SUPERFAMILY) |
| CJ568719 | -8.79 | 6.65E-07 | hypothetical protein | | 451 | 0 | - | | no IPS match |
| TC452171 | -8.72 | 1.14E-06 | ---NA--- | | 227 | 0 | - | | IPR002119 (PRINTS); IPR002119 (SMART); IPR009072 (G3DSA:1.10.20.GENE3D); IPR007125 (PFAM); PTHR23430 (PANTHER); PTHR23430:SF32 (PANTHER); IPR002119 (PROSITE_PATTERNS); IPR009072 (SUPERFAMILY) |
| TC426758 | -8.6 | 8.81E-06 | arginine decarboxylase | | 653 | 7 | P:response to stress; P:response to abiotic stimulus; P:cellular process; P:catabolic process; F:catalytic activity; P:biosynthetic process; P:metabolic process | | no IPS match |
| CD882149 | -8.52 | 5.63E-06 | ---NA--- | | 144 | 0 | - | | no IPS match |
| CA501876 | -8.37 | 1.86E-06 | ---NA--- | | 224 | 0 | - | | SIGNAL_PEPTIDE_H_REGION (PHOBIUS); NON_CYTOPLASMIC_DOMAIN (PHOBIUS); SIGNAL_PEPTIDE_C_REGION (PHOBIUS); SIGNAL_PEPTIDE_N_REGION (PHOBIUS); SIGNAL_PEPTIDE (PHOBIUS); SignalP-TM (SIGNALP_GRAM_POSITIVE); SignalP-noTM (SIGNALP_EUK) |
| TC373881 | -8.15 | 6.9E-06 | eukaryotic initiation factor 4a-2-like | | 1062 | 4 | F:hydrolase activity; F:nucleic acid binding; F:nucleotide binding; P:metabolic process | | G3DSA:2.30.30.50 (GENE3D); IPR003375 (PFAM); IPR003375 (PRODOM); IPR008990 (SUPERFAMILY) |
| CD870828 | -8.15 | 9.73E-06 | hypothetical protein TRIUR3_22538 | | 468 | 0 | - | | no IPS match |
| TC389008 | -8.14 | 8.44E-06 | predicted protein | | 1045 | 0 | - | | no IPS match |
| TC394343 | -7.52 | 9.73E-06 | hypothetical protein TRIUR3_14504 | | 1004 | 0 | - | | NON_CYTOPLASMIC_DOMAIN (PHOBIUS); TRANSMEMBRANE (PHOBIUS); CYTOPLASMIC_DOMAIN (PHOBIUS) |
| TC452354 | -7.46 | 8.81E-06 | ---NA--- | | 402 | 0 | - | | no IPS match |
| TC433360 | -7.21 | 7.87E-06 | 60s ribosomal protein l12 | | 902 | 4 | C:ribosome; F:structural molecule activity; P:translation; P:biological_process | | IPR000558 (PRINTS); IPR000558 (SMART); IPR009072 (G3DSA:1.10.20.GENE3D); IPR007125 (PFAM); IPR000558 (PANTHER); IPR000558 (PROSITE_PATTERNS); IPR009072 (SUPERFAMILY) |
| TC448207 | -7.05 | 1.41E-06 | f-box protein pp2-b10 | | 295 | 4 | P:biosynthetic process; P:carbohydrate metabolic process; P:cellular process; C:membrane | | IPR008207 (SUPERFAMILY) |
| CK169259 | -6.75 | 9.1E-06 | histone h3 | | 791 | 14 | P:transport; C:membrane; C:intracellular; P:biosynthetic process; P:cellular process; C:plastid; F:transferase activity; F:DNA binding; P:cellular component organization; P:DNA metabolic process; F:protein binding; P:biological_process; F:binding; C:nucleus | | IPR012677 (G3DSA:3.30.70.GENE3D); SSF54928 (SUPERFAMILY) |
| TC392698 | -6.45 | 9.31E-06 | elongation factor 1-alpha | | 1185 | 5 | F:translation factor activity, nucleic acid binding; F:nucleotide binding; F:hydrolase activity; C:ribosome; P:translation | | CYTOPLASMIC_DOMAIN (PHOBIUS); NON_CYTOPLASMIC_DOMAIN (PHOBIUS); TRANSMEMBRANE (PHOBIUS); TMhelix (TMHMM) |
| TC458123 | -6.21 | 3.65E-06 | ---NA--- | | 701 | 0 | - | | IPR005067 (PFAM); IPR012348 (G3DSA:1.10.620.GENE3D); PTHR31155:SF2 (PANTHER); PTHR31155 (PANTHER); SIGNAL_PEPTIDE_H_REGION (PHOBIUS); SIGNAL_PEPTIDE_C_REGION (PHOBIUS); NON_CYTOPLASMIC_DOMAIN (PHOBIUS); SIGNAL_PEPTIDE (PHOBIUS); SIGNAL_PEPTIDE_N_REGION (PHOBIUS); SignalP-TM (SIGNALP_GRAM_POSITIVE); IPR009078 (SUPERFAMILY) |
| BQ162513 | -6.17 | 7.61E-06 | ---NA--- | | 437 | 0 | - | | no IPS match |
| TC407339 | -5.85 | 9.73E-06 | metacaspase 2 | | 818 | 6 | C:membrane; C:extracellular region; P:protein metabolic process; P:transport; P:cellular process; F:hydrolase activity | | IPR020472 (PRINTS); IPR001680 (SMART); IPR001680 (PFAM); IPR015943 (G3DSA:2.130.10.GENE3D); PTHR22841 (PANTHER); IPR019775 (PROSITE_PATTERNS); IPR017986 (PROSITE_PROFILES); IPR001680 (PROSITE_PROFILES); IPR001680 (PROSITE_PROFILES); IPR001680 (PROSITE_PROFILES); IPR001680 (PROSITE_PROFILES); IPR017986 (SUPERFAMILY) |
| CA693177 | -5.56 | 2.83E-06 | probable carboxylesterase 12-like | | 335 | 3 | F:hydrolase activity; P:metabolic process; C:plastid | | G3DSA:3.30.497.10 (GENE3D); G3DSA:2.30.39.10 (GENE3D); IPR023796 (PFAM); IPR000215 (PANTHER); IPR023795 (PROSITE_PATTERNS); IPR023796 (SUPERFAMILY) |
| TC402610 | 18.01 | 4.15E-06 | low molecular weight glutenin | | 830 | 1 | F:molecular_function | | no IPS match |
| BJ221811 | 26.94 | 5.41E-07 | disease resistance protein rpm1 | | 639 | 4 | F:hydrolase activity; F:binding; C:plastid; P:nucleobase-containing compound metabolic process | | no IPS match |
| TC391918 | 28.3 | 6.57E-06 | formate dehydrogenase mitochondrial-like | | 1121 | 8 | F:nucleotide binding; F:catalytic activity; C:plastid; C:thylakoid; C:mitochondrion; P:metabolic process; C:cell; P:cellular process | | IPR002653 (SMART); IPR000058 (SMART); IPR002653 (PFAM); IPR000058 (G3DSA:4.10.1110.GENE3D); IPR000058 (PFAM); PTHR10634:SF22 (PANTHER); PTHR10634 (PANTHER); IPR002653 (PROSITE_PROFILES); IPR000058 (PROSITE_PROFILES); SSF118310 (SUPERFAMILY); SSF57716 (SUPERFAMILY) |
| TC393980 | 40.28 | 2.46E-06 | low-molecular-weight glutenin subunit | | 886 | 1 | F:molecular_function | | PTHR22950:SF200 (PANTHER); PTHR22950 (PANTHER); NON_CYTOPLASMIC_DOMAIN (PHOBIUS); SIGNAL_PEPTIDE_N_REGION (PHOBIUS); TRANSMEMBRANE (PHOBIUS); NON_CYTOPLASMIC_DOMAIN (PHOBIUS); TRANSMEMBRANE (PHOBIUS); SIGNAL_PEPTIDE_C_REGION (PHOBIUS); SIGNAL_PEPTIDE (PHOBIUS); SIGNAL_PEPTIDE_H_REGION (PHOBIUS); CYTOPLASMIC_DOMAIN (PHOBIUS); TMhelix (TMHMM); TMhelix (TMHMM); TMhelix (TMHMM) |
| BE413821 | 117.62 | 1.07E-06 | alpha- partial | | 661 | 7 | C:cytoplasm; F:structural molecule activity; F:nucleotide binding; P:cellular process; P:cellular component organization; F:hydrolase activity; C:cytoskeleton | | IPR001878 (SMART); IPR001878 (PFAM); IPR001878 (G3DSA:4.10.60.GENE3D); IPR001878 (PROSITE_PROFILES); IPR001878 (SUPERFAMILY) |
| NP9350187 | 166.51 | 5.41E-07 | low molecular weight glutenin | | 536 | 1 | F:molecular_function | | no IPS match |
| CD915505 | 902.62 | 9.63E-19 | low-molecular-weight glutenin subunit | | 604 | 1 | F:molecular_function | | IPR001954 (PRINTS); IPR001376 (PRINTS); IPR013771 (G3DSA:1.10.120.GENE3D); IPR016140 (PFAM); IPR016140 (SUPERFAMILY) |
| **1b. HNM vs HPBM** Differentially expressed genes identified in the HPBM group when compared to the HNM group. | | | | | | | | | |
| CK162779 | -233.06 | 2.66E-06 | lrr receptor-like serine threonine-protein kinase fls2 | | 1025 | 8 | C:cytoplasm; F:kinase activity; P:cellular protein modification process; F:nucleotide binding; C:membrane; C:plastid; P:metabolic process; P:cellular process | | PR00019 (PRINTS); IPR003591 (SMART); G3DSA:3.80.10.10 (GENE3D); IPR001611 (PFAM); IPR001611 (PFAM); PTHR24420:SF672 (PANTHER); PTHR24420 (PANTHER); SSF52058 (SUPERFAMILY) |
| TC457676 | -180.99 | 4.15E-06 | ---NA--- | | 177 | 0 | - | | TRANSMEMBRANE (PHOBIUS); NON_CYTOPLASMIC_DOMAIN (PHOBIUS); CYTOPLASMIC_DOMAIN (PHOBIUS); TMhelix (TMHMM) |
| CK194194 | -78.27 | 3.13E-17 | fructose-bisphosphate aldolase | | 695 | 13 | P:carbohydrate metabolic process; P:catabolic process; P:response to stress; C:mitochondrion; P:response to abiotic stimulus; P:nucleobase-containing compound metabolic process; C:plastid; C:thylakoid; F:catalytic activity; P:generation of precursor metabolites and energy; P:cellular process; P:biosynthetic process; P:biological_process | | CYTOPLASMIC_DOMAIN (PHOBIUS); TRANSMEMBRANE (PHOBIUS); NON_CYTOPLASMIC_DOMAIN (PHOBIUS); TMhelix (TMHMM) |
| TC450096 | -66.92 | 8.68E-07 | ---NA--- | | 276 | 0 | - | | no IPS match |
| CD897082 | -62.56 | 4.13E-06 | ---NA--- | | 288 | 0 | - | | no IPS match |
| TC392562 | -56.14 | 8.56E-07 | histone h1 | | 1146 | 5 | C:intracellular; F:DNA binding; P:cellular component organization; P:DNA metabolic process; C:nucleus | | no IPS match |
| CK205787 | -53.91 | 1.91E-06 | er lumen protein retaining receptor -like | | 997 | 6 | F:binding; C:cytoplasm; P:cellular process; C:membrane; C:mitochondrion; C:endoplasmic reticulum | | IPR000133 (PRINTS); IPR000133 (PFAM); PTHR10585:SF10 (PANTHER); IPR000133 (PANTHER); CYTOPLASMIC_DOMAIN (PHOBIUS); TRANSMEMBRANE (PHOBIUS); NON_CYTOPLASMIC_DOMAIN (PHOBIUS); NON_CYTOPLASMIC_DOMAIN (PHOBIUS); TRANSMEMBRANE (PHOBIUS); NON_CYTOPLASMIC_DOMAIN (PHOBIUS); CYTOPLASMIC_DOMAIN (PHOBIUS); TRANSMEMBRANE (PHOBIUS); TRANSMEMBRANE (PHOBIUS); TMhelix (TMHMM); TMhelix (TMHMM); TMhelix (TMHMM); TMhelix (TMHMM) |
| CK170902 | -42.71 | 4.71E-06 | udp-glucuronic acid decarboxylase 1-like | | 1081 | 4 | C:cytoplasm; P:metabolic process; F:catalytic activity; F:binding | | IPR001509 (PFAM); G3DSA:3.90.25.10 (GENE3D); PTHR10366 (PANTHER); PTHR10366:SF323 (PANTHER); SSF51735 (SUPERFAMILY) |
| TC448940 | -37.12 | 2.52E-06 | ---NA--- | | 277 | 0 | - | | no IPS match |
| TC375013 | -30.71 | 1.91E-06 | nadh-plastoquinone oxidoreductase subunit k | | 1721 | 12 | C:plasma membrane; F:binding; F:catalytic activity; P:generation of precursor metabolites and energy; P:photosynthesis; C:membrane; C:plastid; C:thylakoid; P:nucleobase-containing compound metabolic process; P:biosynthetic process; P:cellular process; P:transport | | IPR006138 (TIGRFAM); IPR006137 (G3DSA:3.40.50.GENE3D); IPR006137 (PFAM); PTHR11995 (PANTHER); PTHR11995:SF10 (PANTHER); IPR006138 (PROSITE_PATTERNS); SSF56770 (SUPERFAMILY) |
| CK153998 | -29.35 | 5.01E-06 | fiber protein fb34 | | 606 | 2 | C:cytoplasm; C:membrane | | no IPS match |
| TC372980 | -28.54 | 4.75E-22 | 40s ribosomal protein s12 | | 821 | 5 | F:structural molecule activity; C:ribosome; P:translation; C:mitochondrion; P:biological_process | | no IPS match |
| BJ240197 | -28.09 | 6.22E-06 | cytochrome p450 liketbp | | 633 | 0 | - | | CYTOPLASMIC_DOMAIN (PHOBIUS); TRANSMEMBRANE (PHOBIUS); NON_CYTOPLASMIC_DOMAIN (PHOBIUS); CYTOPLASMIC_DOMAIN (PHOBIUS); TRANSMEMBRANE (PHOBIUS); TRANSMEMBRANE (PHOBIUS); NON_CYTOPLASMIC_DOMAIN (PHOBIUS); TMhelix (TMHMM) |
| TC417789 | -24.03 | 6.64E-06 | hypothetical protein F775_17036 | | 1065 | 0 | - | | no IPS match |
| CK196301 | -23.04 | 4.15E-06 | ---NA--- | | 392 | 0 | - | | no IPS match |
| BJ319889 | -22.5 | 2.52E-06 | senescence-associated protein | | 706 | 0 | - | | no IPS match |
| CV769561 | -20.7 | 3.23E-06 | ---NA--- | | 432 | 0 | - | | IPR029039 (G3DSA:3.40.50.GENE3D); IPR029039 (SUPERFAMILY) |
| CK193560 | -20.32 | 4.33E-07 | ribosomal protein l3 | | 456 | 4 | C:ribosome; F:structural molecule activity; P:translation; P:biological_process | | no IPS match |
| TC449147 | -16.18 | 5.01E-06 | ---NA--- | | 286 | 0 | - | | no IPS match |
| TC417298 | -16.08 | 6.2E-06 | 60s ribosomal protein l12 | | 854 | 4 | C:ribosome; F:structural molecule activity; P:translation; P:biological_process | | IPR020784 (G3DSA:3.30.1550.GENE3D); IPR020783 (G3DSA:1.10.10.GENE3D); PTHR11661:SF2 (PANTHER); IPR000911 (PANTHER); IPR020784 (SUPERFAMILY) |
| TC459117 | -15.83 | 1.22E-06 | dihydrolipoyllysine-residue acetyltransferase component 3 of pyruvate dehydrogenase mitochondrial | | 1094 | 11 | C:cytoplasm; F:transferase activity; P:response to stress; C:mitochondrion; C:plastid; P:biosynthetic process; P:carbohydrate metabolic process; P:generation of precursor metabolites and energy; P:catabolic process; P:protein metabolic process; P:cellular process | | no IPS match |
| TC378628 | -11.78 | 6.22E-06 | 40s ribosomal protein s10-like | | 985 | 1 | C:ribosome | | TRANSMEMBRANE (PHOBIUS); NON_CYTOPLASMIC_DOMAIN (PHOBIUS); SIGNAL_PEPTIDE (PHOBIUS); NON_CYTOPLASMIC_DOMAIN (PHOBIUS); TRANSMEMBRANE (PHOBIUS); SIGNAL_PEPTIDE_C_REGION (PHOBIUS); SIGNAL_PEPTIDE_H_REGION (PHOBIUS); SIGNAL_PEPTIDE_N_REGION (PHOBIUS); CYTOPLASMIC_DOMAIN (PHOBIUS); TMhelix (TMHMM); TMhelix (TMHMM); TMhelix (TMHMM) |
| DY741994 | -9.34 | 4.13E-06 | 40s ribosomal protein s21 | | 515 | 6 | P:nucleobase-containing compound metabolic process; F:structural molecule activity; P:translation; C:ribosome; C:cytosol; C:cytoplasm | | no IPS match |
| TC420456 | 6.94 | 6.2E-06 | ac078948_18 serine protease | | 641 | 7 | P:metabolic process; F:catalytic activity; F:protein kinase binding; P:regulation of transcription, DNA-templated; P:regulation of cyclin-dependent protein serine/threonine kinase activity; P:proteolysis; F:peptidase activity | | no IPS match |
| TC375635 | 11.6 | 1.91E-06 | dihydrolipoamide s-acetyltransferase | | 779 | 9 | C:cytoplasm; F:transferase activity; C:mitochondrion; P:biosynthetic process; P:carbohydrate metabolic process; P:generation of precursor metabolites and energy; P:catabolic process; P:protein metabolic process; P:cellular process | | IPR023213 (G3DSA:3.30.559.GENE3D); IPR001078 (PFAM); PTHR23151 (PANTHER); PTHR23151:SF9 (PANTHER); SSF52777 (SUPERFAMILY) |
| CA602991 | 85.03 | 6.94E-08 | inactive poly | | 580 | 2 | F:NAD+ ADP-ribosyltransferase activity; P:metabolic process | | CYTOPLASMIC_DOMAIN (PHOBIUS); TRANSMEMBRANE (PHOBIUS); NON_CYTOPLASMIC_DOMAIN (PHOBIUS) |
| **1c. Common between HNM vs HPAM and HNM vs HPBM** | | | | | | | |  | |
| **Feature ID** | | | | **Seq. Description** | | | |  | |
| BJ240197 | | | | cytochrome p450 liketbp | | | |  | |
| BJ319889 | | | | senescence-associated protein | | | |  | |
| CD897082 | | | | ---NA--- | | | |  | |
| CK153998 | | | | fiber protein fb34 | | | |  | |
| CK162779 | | | | lrr receptor-like serine threonine-protein kinase fls2 | | | |  | |
| TC375013 | | | | nadh-plastoquinone oxidoreductase subunit k | | | |  | |
| TC378628 | | | | 40s ribosomal protein s10-like | | | |  | |
| TC417298 | | | | 60s ribosomal protein l12 | | | |  | |
| TC450096 | | | | ---NA--- | | | |  | |
