# Supplementary material for: Influence of Gene Expression on Hardness in Wheat
Source: PLoS One. 2016 Oct 14;11(10):e0164746. doi: 10.1371/journal.pone.0164746 (PMC5065149; doi:10.1371/journal.pone.0164746)
Supplement: S2 Table — (DOC) [file pone.0164746.s002.doc]

**S2 Table.** Differentially expressed genes identified between the Pin-mutants (HPAM+HPBM) and the non-mutant group (soft+HNM).

| **Feature ID** | **Fold change** | **FDR p-value correction** | **Seq. Description** | **Seq. Length** | #GOs | GOs | InterProScan |
| --- | --- | --- | --- | --- | --- | --- | --- |
| TC378473 | -19.72 | 1.03E-08 | exocyst complex component exo84b-like | 1120 | 4 | C:cytoplasm; P:cellular component organization; P:transport; F:hydrolase activity | no GO terms |
| BJ240197 | -19.99 | 1.09E-08 | cytochrome p450 liketbp | 633 | 0 | - | no GO terms |
| TC415025 | -9.95 | 1.09E-08 | histone h4 | 1281 | 17 | P:protein metabolic process; C:Golgi apparatus; C:nucleolus; C:vacuole; P:nucleobase-containing compound metabolic process; P:transport; C:plasma membrane; C:plastid; P:response to stress; C:thylakoid; F:nucleotide binding; F:DNA binding; P:response to abiotic stimulus; F:protein binding; C:mitochondrion; P:cellular component organization; C:cytosol | no GO terms |
| TC381724 | -33.94 | 1.88E-08 | ferredoxin--nadp leaf isozyme | 1119 | 23 | P:post-embryonic development; P:cellular protein modification process; P:anatomical structure morphogenesis; P:biosynthetic process; C:extracellular region; C:plastid; C:thylakoid; F:nucleotide binding; P:response to biotic stimulus; P:response to abiotic stimulus; F:RNA binding; P:response to external stimulus; P:cell death; F:catalytic activity; P:secondary metabolic process; P:generation of precursor metabolites and energy; P:signal transduction; P:nucleobase-containing compound metabolic process; P:transport; P:response to stress; P:response to endogenous stimulus; P:cellular component organization; P:photosynthesis | no IPS match |
| CA500116 | -17.39 | 1.96E-08 | ---NA--- | 166 | 0 | - | no GO terms |
| CA602991 | 84.74 | 1.96E-08 | inactive poly | 580 | 2 | F:NAD+ ADP-ribosyltransferase activity; P:metabolic process | no GO terms |
| CK153998 | -18.29 | 2.19E-08 | fiber protein fb34 | 606 | 2 | C:cytoplasm; C:membrane | no IPS match |
| TC372980 | -12.62 | 2.19E-08 | 40s ribosomal protein s12 | 821 | 5 | F:catalytic activity; C:mitochondrion; F:structural molecule activity; C:ribosome; P:translation | no IPS match |
| TC456426 | -16.98 | 2.23E-08 | ---NA--- | 637 | 0 | - | no IPS match |
| BE515349 | -99.16 | 2.73E-08 | cytochrome p450 like_tbp | 335 | 1 | F:catalytic activity | no GO terms |
| TC454289 | -19.51 | 2.73E-08 | ---NA--- | 228 | 0 | - | no IPS match |
| TC447207 | -18.69 | 2.73E-08 | ribulose large partial partial | 1648 | 6 | P:carbohydrate metabolic process; F:catalytic activity; P:photosynthesis; P:biosynthetic process; F:binding; C:plastid | nucleic acid binding; nucleotide binding |
| TC451876 | -16.92 | 2.73E-08 | ---NA--- | 513 | 0 | - | no IPS match |
| CA610082 | -14.35 | 2.97E-08 | ---NA--- | 434 | 0 | - | no GO terms |
| TC437369 | -12.72 | 3.51E-08 | nadh dehydrogenase | 622 | 13 | P:protein metabolic process; P:generation of precursor metabolites and energy; P:catabolic process; P:biosynthetic process; P:nucleobase-containing compound metabolic process; P:transport; C:plastid; P:response to stress; P:carbohydrate metabolic process; F:catalytic activity; C:membrane; C:mitochondrion; P:cellular component organization | no IPS match |
| CA498547 | -12.12 | 3.51E-08 | ---NA--- | 249 | 0 | - | no IPS match |
| TC423791 | -8.47 | 3.51E-08 | ---NA--- | 1240 | 0 | - | no GO terms |
| CJ726075 | -65.14 | 4.48E-08 | hypothetical protein TRIUR3_07013 | 761 | 0 | - | no IPS match |
| TC439372 | -11.08 | 6.14E-08 | ---NA--- | 497 | 0 | - | no IPS match |
| CV066181 | 5.84 | 6.14E-08 | alpha beta-gliadin | 734 | 1 | F:molecular_function | nutrient reservoir activity |
| TC441390 | -36.05 | 6.30E-08 | senescence-associated protein | 845 | 1 | C:membrane | no IPS match |
| TC382900 | -9.85 | 6.30E-08 | histone h2a family protein | 863 | 4 | C:nucleus; F:DNA binding; P:biological_process; F:protein binding | nucleus; nucleosome; DNA binding; protein heterodimerization activity |
| TC378628 | -7.89 | 6.30E-08 | 40s ribosomal protein s10-like | 985 | 1 | C:ribosome | no GO terms |
| TC433360 | -6.06 | 7.01E-08 | 60s ribosomal protein l12 | 902 | 3 | F:structural molecule activity; C:ribosome; P:translation | no IPS match |
| TC401729 | -61.81 | 8.06E-08 | rrna intron-encoded homing endonuclease | 1066 | 0 | - | no IPS match |
| TC449482 | -53.2 | 8.06E-08 | cytochrome p450 like_tbp | 1124 | 1 | F:catalytic activity | no IPS match |
| CA668693 | -16.51 | 8.06E-08 | ---NA--- | 466 | 0 | - | no GO terms |
| CK168985 | -9.53 | 8.06E-08 | 60s ribosomal protein l21 | 543 | 4 | C:mitochondrion; F:structural molecule activity; C:ribosome; P:translation | no GO terms |
| CA633111 | -16.42 | 8.14E-08 | ---NA--- | 629 | 0 | - | no IPS match |
| CD892068 | -17.16 | 8.71E-08 | ---NA--- | 667 | 0 | - | no GO terms |
| TC436162 | -25.09 | 9.00E-08 | galactinol--sucrose galactosyltransferase 2 | 477 | 1 | F:catalytic activity | no GO terms |
| TC459382 | -15.68 | 9.00E-08 | lipoxygenase homology domain-containing protein 1-like | 730 | 1 | C:cytoplasm | no IPS match |
| TC374373 | -10.59 | 9.00E-08 | hypothetical protein F775_07910 | 1396 | 2 | F:hydrolase activity; P:metabolic process | no GO terms |
| TC404557 | -15.47 | 9.38E-08 | histone h4 | 656 | 17 | P:protein metabolic process; C:Golgi apparatus; C:nucleolus; C:vacuole; P:nucleobase-containing compound metabolic process; P:transport; C:plasma membrane; C:plastid; P:response to stress; C:thylakoid; F:nucleotide binding; F:DNA binding; P:response to abiotic stimulus; F:protein binding; C:mitochondrion; P:cellular component organization; C:cytosol | no IPS match |
| TC440231 | -12.02 | 9.38E-08 | ---NA--- | 588 | 0 | - | no IPS match |
| TC454154 | -9.8 | 9.84E-08 | disease resistance response protein 206-like | 931 | 1 | C:cytoplasm | no GO terms |
| CA633851 | -17.67 | 1.08E-07 | ---NA--- | 501 | 0 | - | no IPS match |
| TC450485 | -12.73 | 1.14E-07 | ---NA--- | 359 | 0 | - | no IPS match |
| AL820726 | -22.38 | 1.60E-07 | senescence-associated protein | 879 | 0 | - | no GO terms |
| TC435585 | -16.6 | 1.60E-07 | lipoxygenase homology domain-containing protein 1-like | 535 | 1 | C:cytoplasm | no IPS match |
| CA697848 | -11.08 | 1.61E-07 | ---NA--- | 586 | 0 | - | no IPS match |
| AL830834 | -8.38 | 1.73E-07 | histone h2ax-like | 479 | 4 | F:DNA binding; F:protein binding; C:nucleolus; P:nucleobase-containing compound metabolic process | no IPS match |
| TC376511 | -7.93 | 1.73E-07 | 14-3-3 protein | 1091 | 12 | P:catabolic process; P:signal transduction; P:cellular protein modification process; P:biosynthetic process; P:response to stress; P:cell cycle; C:nucleus; F:protein binding; C:cytoplasm; P:lipid metabolic process; F:enzyme regulator activity; F:hydrolase activity | protein domain specific binding |
| TC446772 | -6.2 | 1.73E-07 | ---NA--- | 281 | 0 | - | no IPS match |
| TC413011 | -35.57 | 1.83E-07 | ---NA--- | 828 | 0 | - | no IPS match |
| TC392562 | -20 | 1.83E-07 | histone h1 | 1146 | 3 | C:nucleus; F:DNA binding; P:cellular component organization | no IPS match |
| TC398787 | -6.91 | 1.83E-07 | hypothetical protein F775_05432 | 812 | 0 | - | no IPS match |
| TC414628 | -13.31 | 1.91E-07 | udp-glucuronic acid decarboxylase 6-like isoform x1 | 687 | 3 | C:cytoplasm; F:catalytic activity; F:binding | no GO terms |
| GH725292 | -4.83 | 1.91E-07 | ---NA--- | 573 | 0 | - | no IPS match |
| NP9350187 | 144.19 | 1.91E-07 | low molecular weight glutenin | 536 | 1 | F:molecular_function | nutrient reservoir activity |
| TC455528 | -10.89 | 1.96E-07 | ---NA--- | 228 | 0 | - | no IPS match |
| TC371678 | -6.69 | 1.96E-07 | ---NA--- | 816 | 0 | - | no IPS match |
| CA737330 | -8.88 | 2.03E-07 | ---NA--- | 578 | 0 | - | no IPS match |
| TC393122 | -5.7 | 2.11E-07 | 2-aminoethanethiol dioxygenase-like | 867 | 1 | F:catalytic activity | no IPS match |
| DR731570 | -20.19 | 2.12E-07 | ---NA--- | 288 | 0 | - | no IPS match |
| CA639229 | -16.27 | 2.12E-07 | ---NA--- | 468 | 0 | - | no IPS match |
| AL818129 | -9.04 | 2.12E-07 | ---NA--- | 463 | 0 | - | no GO terms |
| CK197528 | -8.9 | 2.12E-07 | ---NA--- | 255 | 0 | - | no GO terms |
| TC423161 | -12.43 | 2.31E-07 | chlorophyll a-b binding protein cp24 chloroplastic | 700 | 4 | C:membrane; P:photosynthesis; C:plastid; C:thylakoid | no GO terms |
| TC445402 | -5.73 | 2.41E-07 | gamma-glutamyltranspeptidase 1 | 902 | 4 | P:catabolic process; P:lipid metabolic process; F:transferase activity; P:cellular process | gamma-glutamyltransferase activity; glutathione metabolic process |
| CA637743 | -14.53 | 2.47E-07 | ---NA--- | 534 | 0 | - | no IPS match |
| TC451770 | -8.63 | 2.55E-07 | ---NA--- | 255 | 0 | - | no IPS match |
| TC456287 | -8.36 | 2.55E-07 | ---NA--- | 239 | 0 | - | no IPS match |
| TC421004 | -7.16 | 2.55E-07 | predicted protein | 644 | 0 | - | no IPS match |
| TC399832 | -8.78 | 2.58E-07 | beta-expansin 1a precursor | 867 | 6 | C:cytoplasm; C:cell wall; C:membrane; P:cellular component organization; C:extracellular region; P:reproduction | no IPS match |
| TC417298 | -7.73 | 2.58E-07 | 60s ribosomal protein l12 | 854 | 3 | F:structural molecule activity; C:ribosome; P:translation | translation; structural constituent of ribosome; ribosome |
| BJ319889 | -12.13 | 2.74E-07 | senescence-associated protein | 706 | 0 | - | no IPS match |
| TC448029 | -29.5 | 2.79E-07 | proteasome subunit beta type-2-like | 933 | 6 | C:nucleus; P:protein metabolic process; C:cytoplasm; P:catabolic process; P:cellular process; F:hydrolase activity | proteolysis involved in cellular protein catabolic process; proteasome core complex; threonine-type endopeptidase activity |
| TC406029 | -7.42 | 2.79E-07 | beta- partial | 792 | 6 | F:nucleotide binding; C:cytoskeleton; C:cytoplasm; F:structural molecule activity; P:cellular component organization; F:hydrolase activity | no IPS match |
| TC416103 | -12.01 | 2.84E-07 | signal peptidase complex subunit 1 | 576 | 6 | P:protein metabolic process; C:mitochondrion; C:membrane; C:endoplasmic reticulum; P:cellular process; F:hydrolase activity | integral component of membrane; signal peptide processing; signal peptidase complex; peptidase activity |
| BJ234824 | -7.6 | 2.86E-07 | ---NA--- | 319 | 0 | - | no GO terms |
| TC409257 | -6.85 | 2.88E-07 | isopentenyl-diphosphate delta-isomerase chloroplastic | 757 | 4 | P:lipid metabolic process; P:biosynthetic process; P:cellular process; F:hydrolase activity | no IPS match |
| CK167046 | -9.92 | 3.10E-07 | elongation factor 1-alpha | 1165 | 5 | F:nucleotide binding; F:translation factor activity, RNA binding; F:transferase activity; C:ribosome; F:hydrolase activity | no GO terms |
| BG313110 | -6.38 | 3.17E-07 | glycosyltransferase-like domain-containing protein 2-like | 515 | 1 | F:transferase activity | no IPS match |
| TC380174 | -13.77 | 3.33E-07 | senescence-associated protein | 1108 | 1 | C:membrane | no IPS match |
| TC458604 | -107.43 | 3.48E-07 | ---NA--- | 506 | 0 | - | no IPS match |
| TC438213 | -9.79 | 3.48E-07 | ---NA--- | 429 | 0 | - | no IPS match |
| CA680050 | -59.91 | 3.67E-07 | ---NA--- | 636 | 0 | - | no IPS match |
| CA679310 | -8.21 | 3.84E-07 | ---NA--- | 474 | 0 | - | no IPS match |
| TC375013 | -16.28 | 3.88E-07 | nadh-plastoquinone oxidoreductase subunit k | 1721 | 10 | P:generation of precursor metabolites and energy; F:catalytic activity; P:photosynthesis; P:biosynthetic process; F:binding; P:nucleobase-containing compound metabolic process; P:transport; C:plasma membrane; C:plastid; C:thylakoid | oxidation-reduction process; iron-sulfur cluster binding; quinone binding; 4 iron, 4 sulfur cluster binding; NADH dehydrogenase (ubiquinone) activity |
| TC420062 | -7.04 | 3.88E-07 | 60s ribosomal protein l39 | 495 | 4 | C:mitochondrion; F:structural molecule activity; C:ribosome; P:translation | no IPS match |
| GH726561 | -18.66 | 4.39E-07 | t02955probable cytochrome p450 monooxygenase - maize | 580 | 1 | F:catalytic activity | no IPS match |
| TC425360 | -8.89 | 4.76E-07 | 60s ribosomal protein l39 | 700 | 4 | C:mitochondrion; F:structural molecule activity; C:ribosome; P:translation | no GO terms |
| CA641136 | -14.24 | 4.84E-07 | ---NA--- | 263 | 0 | - | no IPS match |
| CA712118 | -30.54 | 4.94E-07 | ---NA--- | 290 | 0 | - | no IPS match |
| TC459152 | -9.04 | 4.98E-07 | ---NA--- | 1129 | 0 | - | no IPS match |
| TC409633 | 80.33 | 5.14E-07 | low molecular weight glutenin | 1463 | 1 | F:molecular_function | nutrient reservoir activity |
| TC425178 | -9.26 | 5.21E-07 | amino acid permease 8-like | 619 | 1 | C:membrane | no IPS match |
| TC381951 | -6.84 | 5.28E-07 | vacuolar proton-inorganic pyrophosphatase | 1109 | 14 | P:generation of precursor metabolites and energy; P:multicellular organismal development; C:Golgi apparatus; F:binding; C:vacuole; P:nucleobase-containing compound metabolic process; C:plasma membrane; C:plastid; F:transporter activity; P:response to stress; P:response to abiotic stimulus; C:endosome; C:mitochondrion; F:hydrolase activity | no IPS match |
| TC454476 | -8.71 | 5.29E-07 | ---NA--- | 185 | 0 | - | no IPS match |
| TC380977 | -8.97 | 5.37E-07 | hypothetical protein F775_31117 | 766 | 0 | - | no IPS match |
| CK162779 | -75.1 | 6.02E-07 | lrr receptor-like serine threonine-protein kinase fls2 | 1025 | 9 | P:cellular protein modification process; F:receptor activity; F:signal transducer activity; C:plasma membrane; C:plastid; F:nucleotide binding; F:protein binding; P:response to endogenous stimulus; F:kinase activity | protein binding |
| CD922604 | -13.01 | 6.10E-07 | ---NA--- | 308 | 0 | - | no GO terms |
| TC434913 | -12.91 | 6.31E-07 | ---NA--- | 404 | 0 | - | no IPS match |
| TC461734 | -13.1 | 6.33E-07 | 26s proteasome non-atpase regulatory subunit 8 | 776 | 2 | P:protein metabolic process; C:intracellular | proteolysis; proteasome regulatory particle |
| TC450096 | -22.55 | 6.53E-07 | ---NA--- | 276 | 0 | - | no IPS match |
| CA606437 | -10.36 | 6.65E-07 | ---NA--- | 431 | 0 | - | no GO terms |
| CD897082 | -29.45 | 6.67E-07 | ---NA--- | 288 | 0 | - | no IPS match |
| TC439212 | -25.52 | 6.67E-07 | cytochrome p450 like_tbp | 813 | 1 | F:catalytic activity | no IPS match |
| TC409288 | -14.51 | 6.67E-07 | hypothetical protein TRIUR3_28366 | 1667 | 1 | C:mitochondrion | no GO terms |
| TC428574 | -13.93 | 6.67E-07 | thiazole biosynthetic enzyme thi4 family | 1081 | 6 | P:carbohydrate metabolic process; P:biosynthetic process; F:binding; C:plastid; P:response to stress; P:cellular process | no IPS match |
| TC417675 | -11.35 | 6.67E-07 | histone -like isoform 2 | 668 | 3 | C:nucleus; F:DNA binding; F:protein binding | no IPS match |
| TC387353 | -10.86 | 6.67E-07 | histone h4 | 700 | 17 | P:protein metabolic process; C:Golgi apparatus; C:nucleolus; C:vacuole; P:nucleobase-containing compound metabolic process; P:transport; C:plasma membrane; C:plastid; P:response to stress; C:thylakoid; F:nucleotide binding; F:DNA binding; P:response to abiotic stimulus; F:protein binding; C:mitochondrion; P:cellular component organization; C:cytosol | nucleus; nucleosome; DNA binding; protein heterodimerization activity; nucleosome assembly |
| TC404223 | -8.14 | 6.67E-07 | hypothetical protein TRIUR3_11328 | 602 | 0 | - | no IPS match |
| TC391063 | -10.52 | 6.69E-07 | atpase subunit 9 | 780 | 8 | F:nucleotide binding; C:membrane; C:mitochondrion; F:lipid binding; P:biosynthetic process; P:nucleobase-containing compound metabolic process; F:transporter activity; F:hydrolase activity | ATP synthesis coupled proton transport; hydrogen ion transmembrane transporter activity; proton-transporting ATP synthase complex, coupling factor F(o); proton-transporting two-sector ATPase complex, proton-transporting domain; ATP hydrolysis coupled proton transport |
| CA609634 | -11.25 | 6.75E-07 | ---NA--- | 460 | 0 | - | no IPS match |
| CA598201 | -8.56 | 6.82E-07 | ---NA--- | 618 | 0 | - | no IPS match |
| TC371080 | -9.61 | 6.92E-07 | beta- partial | 919 | 6 | F:nucleotide binding; C:cytoskeleton; C:cytoplasm; F:structural molecule activity; P:cellular component organization; F:hydrolase activity | structural constituent of cytoskeleton; microtubule-based process; GTP binding; microtubule; GTPase activity |
| TC398986 | -6.71 | 7.23E-07 | disease resistance response protein 206-like | 1005 | 1 | C:cytoplasm | no IPS match |
| TC433638 | -6.17 | 7.26E-07 | ---NA--- | 455 | 0 | - | no IPS match |
| DR735322 | -45.37 | 7.39E-07 | ---NA--- | 1031 | 0 | - | no IPS match |
| TC441874 | -6.41 | 7.39E-07 | pollen-specific leucine-rich repeat extensin-like protein 1-like | 1077 | 2 | F:binding; P:transport | no GO terms |
| TC421603 | -5.61 | 7.39E-07 | polyubiquitin | 901 | 7 | C:nucleus; P:protein metabolic process; F:catalytic activity; P:catabolic process; C:vacuole; P:transport; P:cellular process | protein binding |
| CD454636 | -4.23 | 7.53E-07 | ---NA--- | 506 | 0 | - | no IPS match |
| TC442495 | -9.11 | 8.05E-07 | ---NA--- | 329 | 0 | - | no GO terms |
| TC412273 | -5.47 | 8.05E-07 | subtilisin-like protease sdd1-like | 1614 | 3 | P:protein metabolic process; C:cytoplasm; F:hydrolase activity | no IPS match |
| TC414718 | -9.51 | 8.17E-07 | beta-expansin 1a precursor | 699 | 6 | C:cytoplasm; C:cell wall; C:membrane; P:cellular component organization; C:extracellular region; P:reproduction | no IPS match |
| CA661488 | -8.58 | 8.19E-07 | ---NA--- | 574 | 0 | - | no IPS match |
| CA665155 | -6.37 | 8.40E-07 | 40s ribosomal protein s16-like | 616 | 3 | F:structural molecule activity; C:ribosome; P:translation | no IPS match |
| TC392035 | -6.26 | 8.50E-07 | 40s ribosomal protein s25-1 | 1026 | 1 | C:ribosome | no IPS match |
| TC373881 | -5.42 | 8.80E-07 | eukaryotic initiation factor 4a-2-like | 1062 | 3 | F:nucleic acid binding; F:nucleotide binding; F:hydrolase activity | no GO terms |
| BJ309308 | -19.9 | 8.81E-07 | 60s ribosomal protein l6 | 698 | 3 | F:structural molecule activity; C:ribosome; P:translation | structural constituent of ribosome; ribosome; translation; intracellular |
| TC368687 | -9.54 | 8.81E-07 | apocytochrome b | 2086 | 7 | P:generation of precursor metabolites and energy; F:catalytic activity; C:mitochondrion; C:membrane; F:binding; P:nucleobase-containing compound metabolic process; F:transporter activity | membrane; oxidoreductase activity; electron carrier activity; ubiquinol-cytochrome-c reductase activity; respiratory chain complex III; mitochondrial electron transport, ubiquinol to cytochrome c; respiratory electron transport chain |
| TC405615 | -6.55 | 8.81E-07 | hmg-y-related protein a-like | 963 | 5 | C:nucleus; F:DNA binding; P:cellular component organization; P:biosynthetic process; P:nucleobase-containing compound metabolic process | no IPS match |
| TC389131 | -6.42 | 8.81E-07 | 40s ribosomal protein s10-like | 938 | 1 | C:ribosome | no GO terms |
| TC410644 | -5.28 | 8.81E-07 | fasciclin-like arabinogalactan protein 7 precursor | 1169 | 1 | C:cytoplasm | no IPS match |
| TC430889 | -4.58 | 8.81E-07 | phosphatidate cytidylyltransferase | 480 | 5 | C:membrane; P:lipid metabolic process; P:biosynthetic process; F:transferase activity; P:cellular process | no IPS match |
| TC420043 | 834.7 | 8.81E-07 | hypothetical protein TRIUR3_06809 | 470 | 0 | - | no GO terms |
| TC417789 | -11.38 | 8.85E-07 | hypothetical protein F775_17036 | 1065 | 0 | - | no IPS match |
| TC452538 | -7.95 | 8.97E-07 | ---NA--- | 380 | 0 | - | no GO terms |
| TC389353 | -7.22 | 8.97E-07 | 14 kda proline-rich | 713 | 3 | C:cytoplasm; F:lipid binding; P:transport | no GO terms |
| TC442047 | -9.28 | 8.98E-07 | photosystem ii protein d1 | 1881 | 10 | P:generation of precursor metabolites and energy; F:catalytic activity; C:mitochondrion; C:membrane; P:cellular protein modification process; P:photosynthesis; F:binding; C:plastid; P:response to stress; C:thylakoid | electron transporter, transferring electrons within the cyclic electron transport pathway of photosynthesis activity; photosynthesis, light reaction; photosynthetic electron transport in photosystem II; electron carrier activity |
| TC380921 | -5.86 | 9.21E-07 | 40s ribosomal protein s26 | 733 | 3 | F:structural molecule activity; C:ribosome; P:translation | no IPS match |
| CK172288 | -16.03 | 9.22E-07 | ---NA--- | 921 | 0 | - | no IPS match |
| TC389190 | -7.41 | 9.25E-07 | hmg-y-related protein a-like | 895 | 7 | C:nucleus; F:DNA binding; C:membrane; P:cell differentiation; P:cellular component organization; P:biosynthetic process; P:nucleobase-containing compound metabolic process | no IPS match |
| TC426972 | -17.98 | 9.30E-07 | hypothetical protein F775_28628 | 497 | 0 | - | no IPS match |
| CA698189 | -16.71 | 9.30E-07 | ---NA--- | 490 | 0 | - | no IPS match |
| TC400373 | -8.42 | 9.30E-07 | embr-h2 protein expressed | 653 | 3 | C:cytoplasm; C:membrane; F:binding | no IPS match |
| TC427527 | -7.25 | 9.30E-07 | protein mother of ft and tf 1-like | 843 | 1 | P:biological_process | no GO terms |
| TC377364 | -20.15 | 9.71E-07 | s-adenosylmethionine decarboxylase | 1056 | 3 | F:catalytic activity; P:biosynthetic process; P:nucleobase-containing compound metabolic process | spermine biosynthetic process; spermidine biosynthetic process; adenosylmethionine decarboxylase activity |
| TC453160 | -6.13 | 9.82E-07 | ---NA--- | 540 | 0 | - | no GO terms |
| CV769561 | -9.56 | 0.000001 | ---NA--- | 432 | 0 | - | no GO terms |
| DY741496 | -6.75 | 1.01E-06 | 40s ribosomal protein s16 | 545 | 3 | F:structural molecule activity; C:ribosome; P:translation | no IPS match |
| TC430033 | -6.18 | 1.02E-06 | hypothetical protein TRIUR3_06279 | 545 | 1 | C:cytoplasm | no IPS match |
| TC392043 | -5.89 | 1.02E-06 | amino acid permease 3-like | 918 | 1 | C:membrane | no IPS match |
| TC407153 | -5.82 | 1.02E-06 | histone h4 | 567 | 17 | P:protein metabolic process; C:Golgi apparatus; C:nucleolus; C:vacuole; P:nucleobase-containing compound metabolic process; P:transport; C:plasma membrane; C:plastid; P:response to stress; C:thylakoid; F:nucleotide binding; F:DNA binding; P:response to abiotic stimulus; F:protein binding; C:mitochondrion; P:cellular component organization; C:cytosol | no IPS match |
| CA631441 | -8.96 | 1.03E-06 | gdsl esterase lipase at1g29670-like | 538 | 2 | C:cytoplasm; F:hydrolase activity | no GO terms |
| TC368737 | -7.39 | 1.03E-06 | ethylene-responsive transcription factor rap2-4-like | 1789 | 5 | C:nucleus; F:DNA binding; F:sequence-specific DNA binding transcription factor activity; P:biosynthetic process; P:nucleobase-containing compound metabolic process | no IPS match |
| TC439160 | -8.57 | 1.06E-06 | protein | 1408 | 4 | P:response to abiotic stimulus; C:membrane; C:plastid; P:response to stress | no GO terms |
| GH731673 | -13.92 | 1.07E-06 | zinc finger protein 2 | 700 | 1 | F:metal ion binding | no GO terms |
| TC448847 | -11.09 | 1.07E-06 | cysteine proteinase 1-like | 1122 | 3 | P:protein metabolic process; C:cytoplasm; F:hydrolase activity | proteolysis; cysteine-type peptidase activity |
| CA708173 | -6.26 | 1.07E-06 | receptor-like protein kinase precursor | 508 | 1 | F:kinase activity | no IPS match |
| TC403102 | -5.49 | 1.07E-06 | fasciclin-like arabinogalactan protein 1-like | 1242 | 1 | C:cytoplasmic membrane-bounded vesicle | no GO terms |
| TC446943 | -8.02 | 1.09E-06 | ---NA--- | 473 | 0 | - | no IPS match |
| CA661071 | -7.36 | 1.09E-06 | ---NA--- | 512 | 0 | - | no IPS match |
| TC385207 | -6.78 | 1.09E-06 | histone h2a family protein | 1063 | 4 | C:nucleus; F:DNA binding; P:biological_process; F:protein binding | DNA binding; nucleosome; nucleus; protein heterodimerization activity |
| TC393137 | -5.13 | 1.09E-06 | vamp protein sec22 | 1124 | 0 | - | no GO terms |
| TC413970 | -4.23 | 1.09E-06 | hypothetical protein F775_32693 | 748 | 1 | C:plastid | no IPS match |
| TC421205 | -11.46 | 1.11E-06 | chlorophyll a-b binding protein chloroplastic-like isoform 2 | 532 | 7 | P:generation of precursor metabolites and energy; C:membrane; P:cellular protein modification process; P:photosynthesis; F:binding; C:plastid; C:thylakoid | photosynthesis, light harvesting; membrane |
| CK196988 | -9.42 | 1.11E-06 | ---NA--- | 677 | 0 | - | no IPS match |
| CA690360 | -30.08 | 1.15E-06 | ---NA--- | 442 | 0 | - | protein heterodimerization activity |
| TC401022 | -9.28 | 1.15E-06 | loc100283229 precursor | 755 | 2 | C:cytoplasm; F:molecular_function | electron carrier activity |
| CD891431 | -8.72 | 1.15E-06 | ---NA--- | 323 | 0 | - | no IPS match |
| TC439892 | -16.36 | 1.23E-06 | 50s ribosomal protein l28 | 614 | 7 | P:catabolic process; C:membrane; P:lipid metabolic process; F:structural molecule activity; C:ribosome; C:plastid; P:translation | no GO terms |
| GH728951 | -8.52 | 1.23E-06 | ---NA--- | 692 | 0 | - | no IPS match |
| TC447956 | -5.06 | 1.23E-06 | subtilisin-like protease-like | 708 | 3 | P:protein metabolic process; C:cytoplasm; F:hydrolase activity | no IPS match |
| TC438675 | -5.85 | 1.25E-06 | ---NA--- | 688 | 0 | - | no GO terms |
| TC435042 | -6.02 | 1.30E-06 | ---NA--- | 682 | 0 | - | no GO terms |
| BQ905519 | -15.99 | 1.34E-06 | ---NA--- | 179 | 0 | - | no IPS match |
| CK170902 | -15.46 | 1.36E-06 | udp-glucuronic acid decarboxylase 1-like | 1081 | 1 | C:cytoplasm | no GO terms |
| TC392698 | -4.37 | 1.36E-06 | elongation factor 1-alpha | 1185 | 4 | F:nucleotide binding; F:translation factor activity, RNA binding; C:ribosome; F:hydrolase activity | GTP binding |
| CD882149 | -6.03 | 1.38E-06 | ---NA--- | 144 | 0 | - | no IPS match |
| CK152234 | -5.88 | 1.39E-06 | s-adenosylmethionine decarboxylase 2 | 802 | 3 | F:catalytic activity; P:biosynthetic process; P:cellular process | no IPS match |
| TC429650 | -8.69 | 1.42E-06 | ---NA--- | 407 | 0 | - | no IPS match |
| TC436456 | -21.76 | 1.44E-06 | fructose-bisphosphate aldolase | 570 | 7 | P:carbohydrate metabolic process; P:generation of precursor metabolites and energy; C:cytoplasm; F:catalytic activity; P:catabolic process; P:biosynthetic process; P:nucleobase-containing compound metabolic process | catalytic activity; fructose-bisphosphate aldolase activity; glycolytic process |
| TC444776 | -5.27 | 1.44E-06 | predicted protein | 369 | 0 | - | no IPS match |
| TC413157 | -8.31 | 1.46E-06 | protein transport protein sec61 gamma subunit | 535 | 5 | C:membrane; C:endoplasmic reticulum; F:transporter activity; P:cellular process; C:plastid | no IPS match |
| TC440606 | -7.1 | 1.49E-06 | ---NA--- | 468 | 0 | - | no IPS match |
| TC378765 | -32.84 | 1.52E-06 | ---NA--- | 1061 | 0 | - | no IPS match |
| TC452359 | -8.54 | 1.53E-06 | ---NA--- | 864 | 0 | - | no IPS match |
| TC371466 | -7.23 | 1.60E-06 | ob-fold nucleic acid binding domain containing protein | 972 | 0 | - | no IPS match |
| GH727183 | -6.98 | 1.60E-06 | predicted protein | 719 | 2 | C:mitochondrion; C:membrane | no GO terms |
| TC371597 | -14.37 | 1.63E-06 | low temperature-responsive rna-binding protein | 1020 | 8 | F:nucleotide binding; F:DNA binding; P:response to abiotic stimulus; F:RNA binding; P:response to endogenous stimulus; P:biosynthetic process; P:nucleobase-containing compound metabolic process; P:response to stress | nucleic acid binding; nucleotide binding |
| TC368949 | -30.15 | 1.64E-06 | elongation factor 1-alpha | 1138 | 4 | F:nucleotide binding; F:translation factor activity, RNA binding; F:transferase activity; C:ribosome | no GO terms |
| TC431358 | -4.56 | 1.64E-06 | 60s ribosomal protein l39 | 534 | 4 | C:mitochondrion; F:structural molecule activity; C:ribosome; P:translation | no GO terms |
| TC438437 | -16.13 | 1.65E-06 | senescence-associated protein | 896 | 1 | C:membrane | no GO terms |
| TC444101 | -5.91 | 1.65E-06 | ---NA--- | 549 | 0 | - | no GO terms |
| CK211449 | -19.77 | 1.77E-06 | histone h4 | 695 | 24 | P:multicellular organismal development; P:cellular protein modification process; P:biosynthetic process; C:nucleolus; C:extracellular region; C:vacuole; C:plastid; C:nucleoplasm; C:thylakoid; F:nucleotide binding; F:RNA binding; F:catalytic activity; C:cytosol; P:signal transduction; P:DNA metabolic process; P:transport; C:plasma membrane; P:cell cycle; P:regulation of gene expression, epigenetic; P:response to stress; F:DNA binding; F:protein binding; P:cellular component organization; P:cell differentiation | no GO terms |
| TC458455 | -25.86 | 1.79E-06 | ---NA--- | 169 | 0 | - | no IPS match |
| TC451319 | -6.39 | 1.82E-06 | ---NA--- | 259 | 0 | - | no IPS match |
| TC452342 | -6.2 | 1.93E-06 | ---NA--- | 637 | 0 | - | no IPS match |
| CK199710 | -5.69 | 1.93E-06 | ---NA--- | 717 | 0 | - | no IPS match |
| TC384286 | -6.53 | 2.03E-06 | ob-fold nucleic acid binding domain containing protein | 1025 | 0 | - | no IPS match |
| TC405557 | -5.56 | 2.10E-06 | hypothetical protein | 883 | 1 | C:mitochondrion | no IPS match |
| CK195793 | -12.21 | 2.12E-06 | dihydrolipoyllysine-residue acetyltransferase component of pyruvate dehydrogenase mitochondrial-like | 742 | 2 | C:cytoplasm; P:metabolic process | no IPS match |
| TC390498 | -10.38 | 2.12E-06 | histone h4 | 688 | 17 | P:protein metabolic process; C:Golgi apparatus; C:nucleolus; C:vacuole; P:nucleobase-containing compound metabolic process; P:transport; C:plasma membrane; C:plastid; P:response to stress; C:thylakoid; F:nucleotide binding; F:DNA binding; P:response to abiotic stimulus; F:protein binding; C:mitochondrion; P:cellular component organization; C:cytosol | nucleus; nucleosome; DNA binding; protein heterodimerization activity; nucleosome assembly |
| TC456814 | -7 | 2.12E-06 | histone h2a | 965 | 3 | C:nucleus; F:DNA binding; F:protein binding | DNA binding; nucleus; nucleosome; protein heterodimerization activity |
| TC377027 | -5.79 | 2.12E-06 | 40s ribosomal protein sa-like | 1179 | 5 | F:structural molecule activity; P:cellular component organization; C:cytosol; C:ribosome; P:translation | ribosome; structural constituent of ribosome; intracellular; translation; small ribosomal subunit |
| TC454994 | -5.08 | 2.12E-06 | elongation factor 1-alpha | 1553 | 6 | F:nucleotide binding; F:translation factor activity, RNA binding; F:transferase activity; C:ribosome; P:nucleobase-containing compound metabolic process; F:hydrolase activity | no GO terms |
| BQ242950 | -4.76 | 2.12E-06 | glycosyltransferase-like domain-containing protein 2-like | 344 | 1 | F:transferase activity | no IPS match |
| CK194963 | -11.89 | 2.13E-06 | serine threonine protein phosphatase 2a 59 kda regulatory subunit b eta isoform-like | 705 | 3 | P:signal transduction; F:enzyme regulator activity; C:plastid | protein phosphatase type 2A regulator activity; signal transduction; protein phosphatase type 2A complex; binding |
| TC381825 | -3.89 | 2.14E-06 | hypothetical protein TRIUR3_03969 | 1025 | 12 | P:generation of precursor metabolites and energy; P:catabolic process; P:cellular protein modification process; C:endoplasmic reticulum; C:Golgi apparatus; P:biosynthetic process; C:vacuole; P:nucleobase-containing compound metabolic process; P:carbohydrate metabolic process; F:catalytic activity; C:membrane; P:lipid metabolic process | no GO terms |
| TC448194 | -5.47 | 2.15E-06 | ---NA--- | 425 | 0 | - | no GO terms |
| CD915505 | 94.5 | 2.15E-06 | low-molecular-weight glutenin subunit | 604 | 1 | F:molecular_function | nutrient reservoir activity |
| TC409241 | -6.15 | 2.20E-06 | s-adenosylmethionine decarboxylase | 969 | 3 | F:catalytic activity; P:biosynthetic process; P:nucleobase-containing compound metabolic process | no IPS match |
| TC410710 | -4.42 | 2.27E-06 | 40s ribosomal protein s28 | 1103 | 3 | F:structural molecule activity; C:ribosome; P:translation | no IPS match |
| CK158331 | -14.65 | 2.28E-06 | chlorophyll a-b binding protein | 511 | 7 | P:generation of precursor metabolites and energy; C:membrane; P:cellular protein modification process; P:photosynthesis; F:binding; C:plastid; C:thylakoid | no IPS match |
| CA637539 | -5.41 | 2.28E-06 | ---NA--- | 231 | 0 | - | no IPS match |
| TC428332 | -4.17 | 2.28E-06 | 60s ribosomal protein l24 | 764 | 4 | P:cellular component organization; C:ribosome; F:binding; C:plastid | no IPS match |
| TC448207 | -4.76 | 2.32E-06 | f-box protein pp2-b10 | 295 | 4 | P:carbohydrate metabolic process; C:membrane; P:biosynthetic process; P:cellular process | no GO terms |
| TC407339 | -4.37 | 2.33E-06 | metacaspase 2 | 818 | 5 | P:protein metabolic process; C:membrane; P:transport; P:cellular process; F:hydrolase activity | no IPS match |
| TC446058 | -16.23 | 2.36E-06 | ---NA--- | 706 | 0 | - | no IPS match |
| CK209919 | -6.89 | 2.36E-06 | ---NA--- | 1015 | 0 | - | no GO terms |
| TC417605 | -4.72 | 2.36E-06 | leucine-rich repeat receptor-like serine threonine-protein kinase bam1-like | 877 | 9 | P:cellular protein modification process; F:receptor activity; F:signal transducer activity; C:plasma membrane; F:nucleotide binding; F:protein binding; C:cytoplasm; P:response to endogenous stimulus; F:kinase activity | protein kinase activity; protein phosphorylation; ATP binding |
| TC380702 | -4.23 | 2.46E-06 | fructokinase-2 | 778 | 4 | F:nucleotide binding; P:carbohydrate metabolic process; P:biosynthetic process; F:kinase activity | no GO terms |
| TC416260 | -6.81 | 2.53E-06 | ---NA--- | 659 | 0 | - | no IPS match |
| CK200870 | -8.47 | 2.63E-06 | hypothetical protein F775_32714 | 688 | 0 | - | no IPS match |
| TC460479 | -8.99 | 2.71E-06 | ---NA--- | 737 | 0 | - | no IPS match |
| TC461287 | -20.44 | 2.73E-06 | senescence-associated protein | 638 | 3 | P:oxidation-reduction process; F:oxidoreductase activity; F:aromatase activity | no GO terms |
| TC429054 | -12.46 | 2.73E-06 | ---NA--- | 795 | 0 | - | no IPS match |
| CA629846 | -7.19 | 2.73E-06 | ---NA--- | 500 | 0 | - | no IPS match |
| TC426758 | -6.15 | 2.73E-06 | arginine decarboxylase | 653 | 6 | P:response to abiotic stimulus; F:catalytic activity; P:catabolic process; P:biosynthetic process; P:cellular process; P:response to stress | no GO terms |
| TC433645 | -10.02 | 2.76E-06 | ---NA--- | 540 | 0 | - | no IPS match |
| TC403920 | -19.3 | 2.81E-06 | hypothetical protein F775_32663 | 617 | 0 | - | no IPS match |
| CK195746 | -6.28 | 2.89E-06 | ---NA--- | 441 | 0 | - | no IPS match |
| BQ162609 | -8.02 | 2.93E-06 | ---NA--- | 390 | 0 | - | no IPS match |
| TC385431 | -4.55 | 0.000003 | bag family molecular chaperone regulator 1-like | 729 | 0 | - | no IPS match |
| TC456067 | -33.12 | 3.04E-06 | histone h4-like | 476 | 24 | P:multicellular organismal development; P:cellular protein modification process; P:biosynthetic process; C:nucleolus; C:extracellular region; C:vacuole; C:plastid; C:nucleoplasm; C:thylakoid; F:nucleotide binding; F:RNA binding; F:catalytic activity; C:cytosol; P:signal transduction; P:DNA metabolic process; P:transport; C:plasma membrane; P:cell cycle; P:regulation of gene expression, epigenetic; P:response to stress; F:DNA binding; F:protein binding; P:cellular component organization; P:cell differentiation | no IPS match |
| TC442953 | -14.14 | 3.04E-06 | 60s ribosomal protein l36a | 391 | 3 | F:structural molecule activity; C:ribosome; P:translation | no IPS match |
| TC389492 | -117.98 | 3.05E-06 | hypothetical protein F775_14251 | 922 | 0 | - | no IPS match |
| CJ616829 | -35.74 | 3.07E-06 | senescence-associated protein | 495 | 1 | C:membrane | no IPS match |
| CK164232 | -21.15 | 3.07E-06 | ---NA--- | 275 | 0 | - | no IPS match |
| TC373419 | -5.43 | 3.07E-06 | 40s ribosomal protein s16 | 783 | 3 | F:structural molecule activity; C:ribosome; P:translation | no IPS match |
| TC387680 | -5.35 | 3.07E-06 | fasciclin-like arabinogalactan protein 1-like | 1172 | 1 | F:protein binding | no IPS match |
| TC447291 | -5.18 | 3.07E-06 | PREDICTED: uncharacterized protein LOC100832693 | 1195 | 2 | C:cytoplasm; P:transport | no GO terms |
| TC453569 | -4.72 | 3.07E-06 | ---NA--- | 340 | 0 | - | no IPS match |
| TC402326 | -4.9 | 3.12E-06 | 40s ribosomal protein s16 | 854 | 3 | F:structural molecule activity; C:ribosome; P:translation | ribosome; structural constituent of ribosome; translation |
| CA594384 | -12.1 | 3.43E-06 | ---NA--- | 404 | 0 | - | no IPS match |
| CK197053 | -6.55 | 3.51E-06 | ---NA--- | 732 | 0 | - | no IPS match |
| TC406174 | -8.08 | 3.55E-06 | ---NA--- | 718 | 0 | - | no IPS match |
| TC419057 | -6.26 | 3.61E-06 | histone h2a family protein | 487 | 4 | C:nucleus; F:DNA binding; P:biological_process; F:protein binding | DNA binding; nucleosome; nucleus; protein heterodimerization activity |
| CA628302 | -13.18 | 3.68E-06 | ---NA--- | 307 | 0 | - | no IPS match |
| BJ304521 | -5.56 | 3.68E-06 | unnamed protein product | 357 | 3 | F:nucleotide binding; P:cellular protein modification process; F:kinase activity | no IPS match |
| TC439951 | -17.04 | 3.73E-06 | ---NA--- | 651 | 0 | - | no IPS match |
| TC446162 | -19.33 | 3.85E-06 | histone -like | 842 | 2 | F:binding; C:intracellular | DNA binding; nucleosome; protein heterodimerization activity |
| TC447252 | -10.48 | 3.85E-06 | udp-glucuronic acid decarboxylase 1-like | 1180 | 4 | C:cytoplasmic membrane-bounded vesicle; P:metabolic process; F:catalytic activity; F:coenzyme binding | no IPS match |
| TC368639 | -8.05 | 3.85E-06 | nadh dehydrogenase subunit 4 | 1859 | 8 | P:generation of precursor metabolites and energy; F:catalytic activity; C:membrane; C:mitochondrion; P:photosynthesis; P:biosynthetic process; P:nucleobase-containing compound metabolic process; P:transport | NADH dehydrogenase (ubiquinone) activity; oxidation-reduction process; ATP synthesis coupled electron transport |
| TC438567 | -5.8 | 3.85E-06 | pescadillo homolog | 926 | 8 | F:nucleotide binding; P:protein metabolic process; F:catalytic activity; P:biosynthetic process; C:nucleolus; P:nucleobase-containing compound metabolic process; P:transport; C:nucleoplasm | no GO terms |
| TC422808 | -86.47 | 3.86E-06 | dna-binding protein mnb1b | 967 | 14 | P:generation of precursor metabolites and energy; P:catabolic process; F:structural molecule activity; F:chromatin binding; P:biosynthetic process; P:nucleobase-containing compound metabolic process; P:transport; P:response to stress; C:nucleus; F:DNA binding; P:carbohydrate metabolic process; F:sequence-specific DNA binding transcription factor activity; P:response to abiotic stimulus; P:cellular component organization | no GO terms |
| BQ162513 | -4.11 | 3.86E-06 | ---NA--- | 437 | 0 | - | no IPS match |
| CK216885 | -6.58 | 4.02E-06 | beta- partial | 1086 | 2 | C:cellular_component; P:cellular process | no IPS match |
| TC454701 | -24.16 | 4.08E-06 | ---NA--- | 648 | 0 | - | membrane; photosynthesis, light harvesting |
| TC405912 | -8.34 | 4.08E-06 | harpin-induced family | 754 | 1 | C:mitochondrion | no GO terms |
| TC370292 | -4.54 | 4.08E-06 | structural protein | 1349 | 0 | - | no GO terms |
| CD865470 | -6.22 | 4.09E-06 | kelch repeat-containing f-box-like protein | 335 | 0 | - | no IPS match |
| AL810182 | -6.14 | 4.10E-06 | PREDICTED: uncharacterized protein LOC101752704 | 436 | 2 | C:cytoplasm; P:transport | no GO terms |
| TC378210 | -10 | 4.16E-06 | actin-depolymerizing factor 3 | 847 | 4 | C:cytoskeleton; F:protein binding; C:cytoplasm; P:cellular component organization | intracellular; actin binding; actin cytoskeleton; actin filament depolymerization |
| CK203308 | -35.4 | 4.22E-06 | ---NA--- | 700 | 0 | - | no IPS match |
| DY761206 | -5.65 | 4.28E-06 | ---NA--- | 935 | 0 | - | no IPS match |
| TC454432 | -11.77 | 4.46E-06 | ---NA--- | 223 | 0 | - | no IPS match |
| BG907682 | -43.63 | 4.48E-06 | ferredoxin--nadp leaf chloroplastic | 611 | 3 | F:nucleotide binding; F:catalytic activity; C:plastid | oxidation-reduction process; oxidoreductase activity |
| TC402732 | -38.8 | 4.48E-06 | rrna intron-encoded homing endonuclease | 609 | 0 | - | no IPS match |
| CA694169 | -15.72 | 4.48E-06 | ---NA--- | 648 | 0 | - | no IPS match |
| TC379329 | -4.72 | 4.49E-06 | predicted protein | 1040 | 6 | P:cellular component organization; P:cell differentiation; C:cytosol; P:anatomical structure morphogenesis; P:pollination; P:cell growth | no IPS match |
| TC391915 | -22.48 | 4.61E-06 | histone h4 | 704 | 9 | F:DNA binding; F:protein binding; P:cellular component organization; C:cytosol; C:nucleolus; C:vacuole; C:plasma membrane; C:plastid; C:thylakoid | no IPS match |
| TC408367 | -9.46 | 4.61E-06 | actin-depolymerizing factor 3 | 771 | 4 | C:cytoskeleton; F:protein binding; C:cytoplasm; P:cellular component organization | no IPS match |
| CK198319 | -5.71 | 4.61E-06 | ---NA--- | 743 | 0 | - | no IPS match |
| TC419639 | -5.35 | 4.61E-06 | homoserine kinase | 527 | 4 | F:nucleotide binding; C:mitochondrion; F:kinase activity; C:plastid | no IPS match |
| CD925562 | -4.82 | 4.84E-06 | limonoid udp-glucosyltransferase | 311 | 1 | F:transferase activity | metabolic process; transferase activity, transferring hexosyl groups |
| TC416335 | -5.68 | 4.87E-06 | senescence-associated protein | 1558 | 0 | - | no IPS match |
| TC382796 | -5.54 | 4.87E-06 | ob-fold nucleic acid binding domain containing protein | 1025 | 0 | - | no IPS match |
| TC375979 | -53.45 | 0.000005 | gif1 | 1468 | 11 | P:carbohydrate metabolic process; P:response to biotic stimulus; P:response to external stimulus; C:cytoplasm; C:cell wall; C:endoplasmic reticulum; P:biosynthetic process; C:extracellular region; P:cellular process; P:response to stress; F:hydrolase activity | no GO terms |
| TC407817 | -7.56 | 0.000005 | fiber protein fb34 | 673 | 2 | C:cytoplasm; C:plasma membrane | no IPS match |
| TC458773 | -8.04 | 5.05E-06 | senescence-associated protein | 2127 | 1 | C:membrane | protein folding; calcium ion binding; endoplasmic reticulum; unfolded protein binding; protein binding |
| TC419813 | -12.8 | 5.06E-06 | 14-3-3-like protein | 1176 | 1 | F:protein binding | no IPS match |
| TC395050 | -6.52 | 5.06E-06 | metallothionein-like protein type 2 | 1109 | 2 | C:ribosome; F:binding | no IPS match |
| TC373172 | -4.5 | 5.07E-06 | protein transport protein sec61 subunit alpha-like | 1222 | 3 | C:cytoplasm; C:membrane; P:transport | membrane; protein transport |
| CK211305 | -7.31 | 5.10E-06 | ralfl33 precursor | 758 | 3 | C:cytoplasm; P:nucleobase-containing compound metabolic process; F:hydrolase activity | no IPS match |
| TC416574 | -5.7 | 5.19E-06 | unnamed protein product | 1127 | 0 | - | no IPS match |
| TC395672 | -13.31 | 5.28E-06 | nadh dehydrogenase subunit 1 | 666 | 7 | P:generation of precursor metabolites and energy; F:catalytic activity; C:mitochondrion; P:biosynthetic process; P:nucleobase-containing compound metabolic process; P:transport; C:plasma membrane | oxidation-reduction process; membrane |
| TC443019 | -11.92 | 5.30E-06 | ---NA--- | 579 | 0 | - | no IPS match |
| TC410807 | -6.03 | 5.42E-06 | beta-expansin 1a expressed | 1282 | 6 | C:cytoplasm; C:cell wall; C:membrane; P:cellular component organization; C:extracellular region; P:reproduction | no IPS match |
| TC379959 | -3.49 | 5.46E-06 | elongation factor 1-alpha | 1127 | 6 | F:nucleotide binding; F:translation factor activity, RNA binding; F:transferase activity; C:ribosome; P:nucleobase-containing compound metabolic process; F:hydrolase activity | no GO terms |
| TC459101 | -16.37 | 5.48E-06 | photosystem ii protein m | 793 | 6 | P:generation of precursor metabolites and energy; C:mitochondrion; C:membrane; P:photosynthesis; C:plastid; C:thylakoid | no GO terms |
| TC413901 | -5.21 | 5.48E-06 | phytosulfokine receptor 1-like | 753 | 4 | F:nucleotide binding; C:membrane; P:cellular protein modification process; F:kinase activity | no IPS match |
| CA694612 | -5 | 5.48E-06 | ---NA--- | 497 | 0 | - | no IPS match |
| CA658950 | -3.97 | 5.61E-06 | ---NA--- | 298 | 0 | - | no GO terms |
| CA680158 | -9.47 | 5.65E-06 | ---NA--- | 691 | 0 | - | no IPS match |
| TC428668 | -5.88 | 5.82E-06 | osmotin-like protein | 500 | 6 | C:cytoplasm; C:cell wall; P:cellular component organization; P:biosynthetic process; C:extracellular region; P:cell cycle | no IPS match |
| TC426484 | -10.25 | 5.83E-06 | ---NA--- | 461 | 0 | - | no IPS match |
| TC438636 | -7.86 | 5.89E-06 | mitochondrial phosphate carrier protein mitochondrial-like | 953 | 10 | P:carbohydrate metabolic process; P:response to abiotic stimulus; P:catabolic process; C:cell wall; C:membrane; C:vacuole; P:nucleobase-containing compound metabolic process; P:transport; C:plastid; P:response to stress | no GO terms |
| TC404642 | -3.59 | 5.94E-06 | signal peptidase complex subunit 1 | 746 | 6 | P:protein metabolic process; C:mitochondrion; C:membrane; C:endoplasmic reticulum; P:cellular process; F:hydrolase activity | signal peptidase complex; peptidase activity; integral component of membrane; signal peptide processing |
| CA627482 | -12.64 | 5.95E-06 | ---NA--- | 474 | 0 | - | no IPS match |
| TC422097 | -6.09 | 5.95E-06 | alpha- partial | 795 | 5 | F:nucleotide binding; C:cytoskeleton; F:structural molecule activity; P:cellular component organization; F:hydrolase activity | no IPS match |
| TC405324 | -10.18 | 6.23E-06 | actin-depolymerizing factor 3 | 891 | 4 | C:cytoskeleton; F:protein binding; C:cytoplasm; P:cellular component organization | intracellular; actin binding; actin cytoskeleton; actin filament depolymerization |
| TC449355 | -8.59 | 6.30E-06 | ---NA--- | 277 | 0 | - | no IPS match |
| TC459397 | -4.27 | 6.56E-06 | predicted protein | 468 | 1 | F:DNA binding | no GO terms |
| TC411797 | -5.66 | 6.57E-06 | qltg3-1 | 1333 | 2 | C:cytoplasm; P:response to stress | defense response |
| TC387710 | -5.2 | 6.57E-06 | u6 snrna-associated sm-like protein lsm3-like | 656 | 3 | P:DNA metabolic process; C:intracellular; P:response to stress | no GO terms |
| TC376416 | -4.26 | 6.57E-06 | histone h3 | 1185 | 9 | C:nucleus; F:DNA binding; P:carbohydrate metabolic process; F:protein binding; P:catabolic process; F:structural molecule activity; C:ribosome; P:transport; P:translation | no IPS match |
| TC395918 | -8.36 | 6.62E-06 | s-adenosylmethionine decarboxylase | 770 | 3 | F:catalytic activity; P:biosynthetic process; P:nucleobase-containing compound metabolic process | no IPS match |
| TC444065 | -7.82 | 6.83E-06 | protein tar1 | 296 | 1 | C:membrane | no GO terms |
| CA635877 | -15.46 | 6.88E-06 | ---NA--- | 631 | 0 | - | no IPS match |
| TC452171 | -5.23 | 6.88E-06 | ---NA--- | 227 | 0 | - | no IPS match |
| CA670594 | -4.68 | 6.88E-06 | ---NA--- | 187 | 0 | - | no GO terms |
| TC373370 | -3.92 | 6.88E-06 | subtilisin-like protease | 1185 | 3 | P:protein metabolic process; C:cytoplasm; F:hydrolase activity | no IPS match |
| TC408656 | -3.2 | 6.88E-06 | proteasome subunit alpha type-4 | 852 | 6 | C:nucleus; P:protein metabolic process; P:catabolic process; C:mitochondrion; P:cellular process; F:hydrolase activity | proteolysis involved in cellular protein catabolic process; proteasome core complex; threonine-type endopeptidase activity |
| CA648080 | -10.08 | 0.000007 | ---NA--- | 239 | 0 | - | no IPS match |
| CD896526 | -6.53 | 0.000007 | ---NA--- | 189 | 0 | - | no IPS match |
| CA652472 | -6.84 | 7.14E-06 | ---NA--- | 289 | 0 | - | no GO terms |
| TC428354 | -4.73 | 7.14E-06 | hypothetical protein F775_16832 | 440 | 0 | - | no IPS match |
| TC439414 | -14.88 | 7.52E-06 | elongation factor 1-delta 1 | 559 | 1 | P:translation | no IPS match |
| TC385772 | -10.66 | 7.63E-06 | defensin precursor | 705 | 2 | C:cytoplasm; P:response to stress | no IPS match |
| TC449147 | -8.07 | 7.63E-06 | ---NA--- | 286 | 0 | - | no IPS match |
| CK216375 | -9.18 | 7.67E-06 | zf-hd homeobox protein at4g24660-like | 1019 | 2 | F:metal ion binding; C:cytoplasm | no IPS match |
| TC431716 | -5.7 | 7.71E-06 | chlorophyll a b-binding protein | 1060 | 7 | P:generation of precursor metabolites and energy; C:membrane; P:cellular protein modification process; P:photosynthesis; F:binding; C:plastid; C:thylakoid | no IPS match |
| TC448646 | -4.3 | 7.71E-06 | tpa: leucine-rich repeat family protein | 330 | 2 | C:cytoplasm; F:kinase activity | no IPS match |
| TC450757 | -6.52 | 7.72E-06 | hypothetical protein F775_31117 | 1028 | 0 | - | no GO terms |
| TC410983 | -5.72 | 8.02E-06 | elongation factor 1-alpha | 1023 | 6 | F:nucleotide binding; F:translation factor activity, RNA binding; F:transferase activity; C:ribosome; P:nucleobase-containing compound metabolic process; F:hydrolase activity | GTP binding |
| CK195773 | -4.31 | 8.13E-06 | transcription elongation factor a protein 2 | 703 | 5 | C:nucleus; F:DNA binding; F:translation factor activity, RNA binding; C:ribosome; P:nucleobase-containing compound metabolic process | zinc ion binding; transcription, DNA-templated; nucleic acid binding |
| TC460561 | -6.07 | 8.17E-06 | ---NA--- | 542 | 0 | - | no IPS match |
| GH731500 | -5.44 | 8.17E-06 | metacaspase 2 | 567 | 5 | P:protein metabolic process; C:membrane; P:transport; P:cellular process; F:hydrolase activity | no IPS match |
| CD870828 | -5.11 | 8.17E-06 | hypothetical protein TRIUR3_22538 | 468 | 0 | - | no IPS match |
| CA635213 | -8.74 | 8.44E-06 | ---NA--- | 641 | 0 | - | no IPS match |
| TC416021 | -7.81 | 8.55E-06 | subtilisin-like protease sdd1-like | 1390 | 3 | P:protein metabolic process; C:cytoplasm; F:hydrolase activity | proteolysis; serine-type endopeptidase activity |
| TC385791 | -6.55 | 8.70E-06 | fasciclin-like arabinogalactan protein 1-like | 1053 | 1 | C:cytoplasm | no IPS match |
| TC448500 | -4.56 | 8.70E-06 | nuclear transcription factor y subunit b-3 | 1240 | 6 | C:nucleus; F:DNA binding; F:sequence-specific DNA binding transcription factor activity; F:protein binding; P:biosynthetic process; P:nucleobase-containing compound metabolic process | no GO terms |
| TC413253 | -7.17 | 8.72E-06 | ferredoxin-thioredoxin variable chain-like | 535 | 6 | P:lipid metabolic process; P:photosynthesis; P:biosynthetic process; F:transferase activity; F:binding; C:plastid | no IPS match |
| TC453463 | -7.01 | 8.72E-06 | ---NA--- | 601 | 0 | - | no IPS match |
| CJ552559 | -6.16 | 8.72E-06 | hypothetical protein TRIUR3_05784 | 531 | 0 | - | no IPS match |
| CA711962 | 14.06 | 8.72E-06 | chymotrypsin inhibitor-2 | 501 | 5 | P:protein metabolic process; C:extracellular region; F:enzyme regulator activity; P:response to stress; P:cellular process | response to wounding; serine-type endopeptidase inhibitor activity |
| CJ870951 | -8.57 | 8.75E-06 | ---NA--- | 282 | 0 | - | no IPS match |
| TC384454 | -7.48 | 8.81E-06 | histone h3 | 695 | 5 | C:nucleus; F:DNA binding; P:biological_process; F:protein binding; C:plastid | no GO terms |
| CK160307 | -16.27 | 9.04E-06 | ---NA--- | 363 | 0 | - | no IPS match |
| TC441531 | -6.53 | 9.39E-06 | ---NA--- | 426 | 0 | - | no IPS match |
| TC449358 | -11.41 | 9.53E-06 | chk1 checkpoint-like partial | 852 | 0 | - | no IPS match |
| TC369379 | -5.71 | 9.56E-06 | elongation factor 1-alpha | 1149 | 6 | F:nucleotide binding; F:translation factor activity, RNA binding; F:transferase activity; C:ribosome; P:nucleobase-containing compound metabolic process; F:hydrolase activity | no GO terms |
| TC396202 | -3.88 | 9.56E-06 | PREDICTED: uncharacterized protein LOC100832693 | 773 | 2 | C:cytoplasm; P:transport | no GO terms |
| CK159199 | -116.63 | 9.66E-06 | aldehyde oxidase 4 | 651 | 6 | F:nucleotide binding; F:catalytic activity; P:catabolic process; C:cytosol; P:nucleobase-containing compound metabolic process; C:plastid | no GO terms |
| GH725133 | -28.13 | 9.66E-06 | ---NA--- | 645 | 0 | - | no IPS match |
| TC407625 | -8.71 | 9.66E-06 | calmodulin-related protein touch-induced | 590 | 1 | F:binding | no IPS match |
| TC442858 | -5.27 | 9.68E-06 | ---NA--- | 372 | 0 | - | no IPS match |
| TC427874 | -5.19 | 9.73E-06 | nac transcription factor | 573 | 4 | C:nucleus; F:DNA binding; P:biosynthetic process; P:nucleobase-containing compound metabolic process | no IPS match |
| TC417284 | -7.54 | 9.93E-06 | rrna intron-encoded homing endonuclease | 547 | 0 | - | no GO terms |
