# Supplementary material for: Influence of Gene Expression on Hardness in Wheat
Source: PLoS One. 2016 Oct 14;11(10):e0164746. doi: 10.1371/journal.pone.0164746 (PMC5065149; doi:10.1371/journal.pone.0164746)
Supplement: S3 Table — Duplicates were grown in a glasshouse and analysed to exmine reprodctibility of expression patterns. (DOC) [file pone.0164746.s003.doc]

**S3 Table. *Pin* gene expression results for duplicate experiments for the same genotypes at 14 DPA.** Duplicates were grown in a glasshouse and analysed to examine reproducibility of expression patterns.

|  | 14 DPA | | | |
| --- | --- | --- | --- | --- |
|  |  |  | Duplicate | |
| **Genotype** | ***Pina* (RPKM)** | ***Pinb* (RPKM)** | ***Pina* (RPKM)** | ***Pinb* (RPKM)** |
| Banks | 7756 | 7280 | 9715 | 5364 |
| Ellison | 2 | 2930 | 73 | 2789 |
| Gabo | 2 | 2885 | 16 | 4873 |
| Gregory | 7164 | 3158 | 9273 | 5738 |
| Kite | 13172 | 5627 | 8647 | 5670 |
| Sunco | 14291 | 4677 | 9807 | 6142 |
